# Single-cell atlas of the esophageal squamous cell carcinoma immune ecosystem to predict immunotherapy response

**DOI:** 10.1038/s41392-025-02446-x

**Published:** 2025-10-20

**Authors:** Xiankai Chen, Yahui Zhao, Yuhao Wang, Xiliang Wang, Yuhao Liu, Zhihua Liu, Yin Li

**Affiliations:** 1 Section of Esophageal and Medical Oncology, Department of Thoracic Surgery, National Cancer Center/National Clinical Research Center for Cancer/Cancer Hospital, Chinese Academy of Medical Sciences and Peking Union Medical College, Beijing, China; 2 State Key Laboratory of Molecular Oncology, National Cancer Center, National Clinical Research Center for Cancer, Cancer Hospital, Chinese Academy of Medical Sciences and Peking Union Medical College, Beijing, China; 3 Institute of Cancer Research, Henan Academy of Innovations in Medical Sciences, Zhengzhou, Henan Province China

**Keywords:** Cancer microenvironment, Gastrointestinal cancer, Cancer therapy, Tumour immunology

## Abstract

Esophageal squamous cell carcinoma (ESCC) evolves within a highly interactive tumor microenvironment (TME) that shapes therapeutic response. We utilized mass cytometry to analyze over 10 million cells from 25 ESCC tumors, 24 adjacent nontumor tissues, and 23 peripheral blood samples, employing an extensive panel of 42 immune markers. The resulting atlas reveals a compartmentalized landscape with a reproducible paucity of CD4⁺ and CD8⁺ central memory T cells (TCM) in tumor sites. Reintroduction of patient-derived TCMs restored antitumor immunity in coculture assays, demonstrating their cytotoxic capacity in vitro and suggesting their potential relevance for future therapeutic exploration. Myeloid profiling identified PD-L1⁺ tumor-associated macrophages (TAMs) as correlates of clinical benefit; ex vivo PD-L1 blockade reprogrammed TAMs toward proinflammatory states, indicating pharmacological malleability. Notably, CD39⁺ tumor-infiltrating T cells were consistently associated with favorable prognosis and increased responsiveness to PD-1 blockade across cancer types. The functional inhibition of CD39 impaired cytotoxic T-cell activity, underscoring its dual role as a marker of immune dysfunction and a promising therapeutic target. Collectively, our findings provide a comprehensive immune landscape of ESCC, highlighting key immunological deficits and opportunities for targeted interventions. The insights gained underscore the potential of tailoring immunotherapies to the specific immune profiles of the TME, potentially revolutionizing treatment paradigms for ESCC patients. This study sets the stage for a more nuanced understanding and manipulation of the immune elements critical for optimizing cancer immunotherapy.

## Introduction

Esophageal cancer imposes a substantial global burden, with disproportionately high incidence and mortality in East Asia. According to the latest statistics, it ranks seventh for incidence and sixth for cancer deaths worldwide.^1^ The highest-risk regions include Asia and East Africa, where China alone accounted for more than 224,012 new cases in 2022.^2^ Two principal histological subtypes are recognized—esophageal squamous cell carcinoma (ESCC) and esophageal adenocarcinoma (EAC).^3,4^ In China, ESCC is the predominant subtype, accounting for more than 90% of all esophageal cancer cases. ESCC predominates (≥90% of cases) and is often detected at advanced stages, reflecting rapid local invasion and early dissemination; accordingly, outcomes remain poor, with 5-year survival ~15–25%.^5,6^ Beyond stage at diagnosis, lifestyle and environmental exposures (including tobacco, alcohol, and dietary nitrosamines) as well as region-specific socioeconomic factors likely shape disease risk and late presentation.^2^ Screening and early detection programs remain uneven, and for patients with advanced disease, durable therapeutic options are still limited. These realities underscore the urgency of refining disease taxonomy and translating biological insights into actionable strategies for risk stratification and treatment selection, with particular relevance for high-burden settings such as China.

ESCC displays extensive genomic and epigenomic diversity that fosters phenotypic plasticity, clonal evolution and treatment resistance. Dissecting this complexity requires high-dimensional approaches capable of resolving cell states and spatial organization at single-cell resolution.^7^ Recent applications of single-cell RNA sequencing, cytometry by time-of-flight (CyTOF), and multiplex immunohistochemistry (mIHC) have begun to delineate a more granular atlas of the ESCC tumor microenvironment (TME).^8–10^ These studies converge on a composite landscape featuring exhausted CD8⁺ T cells, FOXP3⁺ regulatory T cells (Tregs), and alternatively activated macrophages that collectively promote immune escape. A detailed immune cell atlas has been constructed, revealing that CD8^+^ T cells transition from preexhausted to exhausted states, reflecting a dynamic yet ultimately suppressed anti-tumor response.^8^ Furthermore, the presence of immunosuppressive cells and factors within the TME has been linked to poor clinical outcomes, highlighting the need for targeted immunotherapeutic strategies.^11^ In parallel, spatially resolved profiling has emphasized that not only the abundance but also the geographic context of immune and stromal populations influences function, antigen exposure and response to therapy. Together, converging evidence links these cellular circuits to inferior outcomes, catalyzing a shift from one-size-fits-all immunotherapy to rational, biomarker-guided strategies that account for patient-specific immune ecologies.^12–15^

Within this framework, two axes have attracted particular attention in ESCC and related squamous malignancies. First, tumor-associated macrophages (TAMs) frequently express PD-L1, and their abundance and activation state appear to modulate responses to PD-(L)1 blockade. Emerging studies indicate that PD-L1 in myeloid cells functions less as a static label and more as a tunable rheostat: PD-L1 ligation and its interruption can reprogram macrophage polarization toward proinflammatory states, suggesting that the myeloid context should inform checkpoint selection, sequencing and combinations.^16–18^ Second, CD39 (ENTPD1)—an ectonucleotidase induced by chronic T-cell stimulation—enriches for tumor-reactive lymphocytes across solid tumors, particularly when coexpressed with CD103. In ESCC and related squamous cancers, elevated CD39 on tumor-infiltrating T cells correlates with improved survival and responsiveness to PD-1 blockade; perturbational data further show that pharmacologic inhibition of CD39 can blunt IFN-γ/GZMB programs and cytotoxic function, underscoring a context-dependent role as both a readout of tumor reactivity and a regulator of adenosinergic immunosuppression.^19–21^ Complementing these axes is memory biology: ESCC tumors often exhibit a relative paucity of central memory T (TCM) cells, and replenishing this compartment—in preclinical autologous settings—can restore durable, recall-competent cytotoxicity. Conceptually, these lines of evidence argue for integrative profiling that links cell-state identity to function and clinical endpoints, so as to develop composite biomarkers and combination regimens that align with a patient’s innate immune ecology.

Against this backdrop, we conducted a detailed investigation into the immune landscape of ESCC and its relationship with prognostic outcomes. We profiled immune cells from the blood, normal tissue, and tumor tissue from 25 ESCC patients using CyTOF, and integrated these high-dimensional proteomic profiles with scRNA-seq to gain a deeper understanding of the tumor microenvironment. Spatially resolved mIHC was further employed to map the spatial organization of key immune populations within the tumor. This integrative approach was designed to address three key questions: (i) which compartment-specific cell states and spatial arrangements are recurrent across patients; (ii) whether CD39^high^ T-cell populations and PD-L1^+^ TAMs capture response-relevant biology; and (iii) whether functional deficits in the memory pool can be mitigated by targeted replenishment strategies. Our analyses reveal a reproducible architecture in which exhausted CD8^+^ T cells interdigitate with immunosuppressive myeloid populations; PD-L1^+^ TAMs are reprogrammable by checkpoint blockade and track with clinical benefit; CD39-expressing tumor-infiltrating T cells are enriched in responders and associate with superior survival; and supplementation of TCM restores antitumor cytotoxicity in autologous systems. Together, these findings provide a mechanistically anchored framework for patient stratification and for designing combinations that extend the benefits of immunotherapy in ESCC.

## Results

### Single-cell proteomic landscape of major immune cell types from ESCC patients

**Fig. 1:**
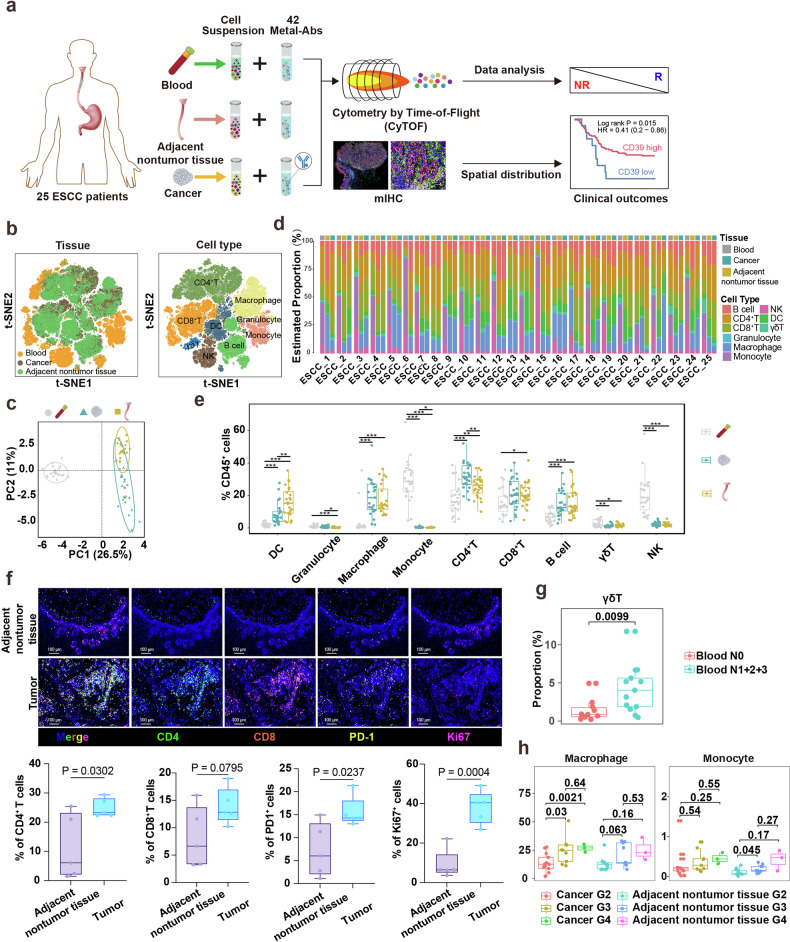
Proteomic atlas of major immune cells in human ESCC. **a** Overview of study workflow: specimen collection, CyTOF profiling (42-marker panels), computational analysis and validation. **b** t-SNE map of the major immune cell types and their origins in ESCC patients. **c** PCA plot presenting the mean expression of 42 selected markers in each ESCC patient. Points with varying shapes and colors represent different tissue groups. **d** Stacked bar plots presenting major immune cell frequencies in the blood, adjacent nontumor tissue and tumor tissue of patients with ESCC. **e** Box-and-whisker plots showing the distribution of major immune cell subpopulations across ESCC tissue origins. Centre line, median; box, interquartile range; whiskers, 1.5× IQR. Kruskal–Wallis test with Benjamini–Hochberg post hoc correction. *P* < 0.05 (*), *P* < 0.01 (**), *P* < 0.001 (***). **f** Multiplex IHC was used to explore the spatial distribution of immune cells within ESCC tissues. mIHC of PD-1 (yellow), CD4 (green), Ki67 (magenta), and CD8 (red). Scale bars, 100 μm. **g** Box-and-whisker plot of γδ T-cell abundance by lymph-node status. Mann‒Whitney *U* test. **h** B cells were significantly elevated in advanced tumors (G3 and G4). Kruskal–Wallis test with Benjamini–Hochberg post hoc correction

To chart immune diversity and phenotype in the ESCC tumor microenvironment and explore their potential clinical relevance, we performed CyTOF profiling of freshly resected tumors, matched adjacent nontumor tissue and peripheral blood samples from 25 ESCC patients, as schematized in Fig. 1a; patient characteristics in Supplementary Table 1. We employed a comprehensive 42-antibody panel to characterize major immune cell populations and their states of activation and exhaustion across different tissue compartments (Fig. 1a). For efficient processing, we utilized tag-based barcoding^22^ to aggregate single-cell suspensions from all collected samples prior to staining, thus facilitating high-throughput and consistent examination of the ESCC TME. This approach enabled us to systematically explore the complex interactions within the TME that may influence patient prognosis and response to therapy.

We performed CyTOF profiling and isolated approximately 10,000 immune cells (CD45^+^ CD66b^-^) per sample from the blood, adjacent nontumor tissue, and tumor tissues of ESCC patients, allowing for an in-depth analysis of the TME composition. We processed high-dimensional cytometry data to examine the immune cell profiles across these samples. Our analysis revealed significant commonalities in immune response mechanisms among the 25 ESCC patients, as demonstrated by the overlap in immune cell profiles (Fig. 1b left panel, Supplementary Figs. 1a, 2a). We analyzed aggregated CyTOF data to identify 53 distinct immune cell clusters within our ESCC patient cohort. Unsupervised clustering with lineage-defining markers resolved nine broad leukocyte classes: CD4⁺ T, CD8⁺ T, γδ T, B, natural killer (NK), dendritic cells (DC), granulocytes, macrophages and monocytes (Fig. 1b right panel, Supplementary Figs. 1b, 2b). At the compartment level, tumor and adjacent nontumor tissues exhibited closely matched immune compositions, whereas peripheral blood displayed a distinct profile (Fig. 1c). This divergence reflected shifts across dendritic cells, granulocytes, macrophages/monocytes and lymphoid subsets (CD4⁺ T, B, γδ T and NK cells), with selected monocytic populations—including immunoregulatory phenotypes—relatively enriched in circulation (Fig. 1d, e). To provide spatial context, multiplex IHC demonstrated a higher intratumoral density of CD4⁺ T cells than in adjacent tissue (Fig. 1f), consistent with a localized immune response that may shape ESCC biology and clinical outcome.

Compared with node-negative cases, patients with lymph-node metastasis showed a significant enrichment of circulating γδ T cells, nominating this subset as a candidate peripheral biomarker of metastatic burden and a putative contributor to systemic immune responses during tumor dissemination (Fig. 1g). Moreover, we noted that the infiltration of macrophages in the tumor tissues of G4 and G3 ESCC patients was significantly greater than that in G2 ESCC patients. This observation suggests a correlation between the degree of malignancy and macrophage infiltration, with a potential role for these cells in promoting tumor progression (Fig. 1h).

### Treg-enriched CD4⁺ T-cell phenotypes in the ESCC TME

**Fig. 2:**
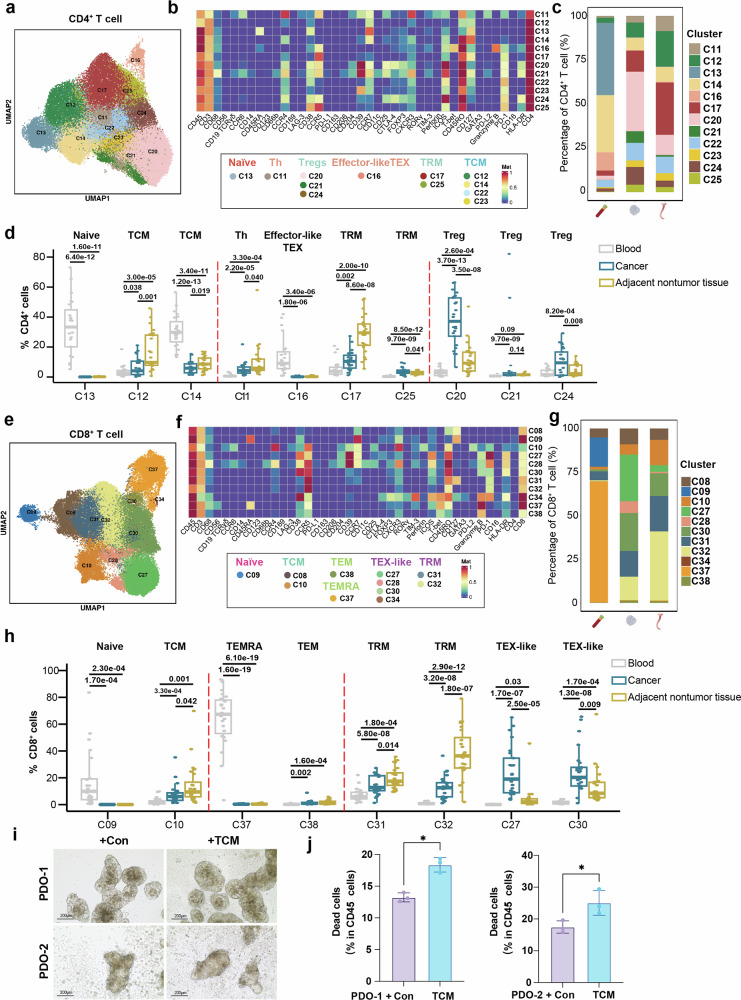
Characterizing T-cell phenotypes and TCM antitumor strategies in the ESCC TME. **a** Uniform manifold approximation and projection (UMAP) showing the distribution of CD4^+^ T-cell subpopulations in ESCC patients. **b** Heatmap showing the average expression levels of representative protein markers in CD4^+^ T-cell subpopulations. **c** Stacked bar plots presenting the frequencies of major CD4^+^ T cells in the blood, adjacent nontumor and tumor tissues of patients with ESCC. **d** Box-and-whisker plots showing the distribution of CD4^+^ T-cell subpopulations in patients with different origins of ESCC. Kruskal–Wallis test with Benjamini–Hochberg post hoc correction. **e** UMAP plot showing the distribution of CD8^+^ T-cell subpopulations in ESCC patients. **f** Heatmap showing the average expression levels of representative protein markers in CD8^+^ T-cell subpopulations. **g** Sankey diagram presenting the frequencies of major CD8^+^ T cells in the blood, normal and tumor tissues of patients with ESCC. **h** Box-and-whisker plots showing the distribution of CD8^+^ T-cell subpopulations in patients with different origins of ESCC. Kruskal–Wallis test with Benjamini–Hochberg post hoc correction. **i** Bright-field images of PDO-1 and PDO-2 cultured alone or cocultured with autologous CD62L⁺ TCM cells for 5 days. Organoids cocultured with TCM cells are reduced in size and structural integrity, indicating T-cell-mediated cytotoxicity. Scale bars, 200 μm. **j** Quantification of dead tumor cells (annexin V⁺/PI⁺ in the CD45⁻ population) in the PDO-1 and PDO-2 coculture systems. The data are shown as the means ± SDs (*n* = 3). Two-tailed t test; *P* < 0.05 (*)

We identified 12 distinct CD4^+^ T-cell clusters in ESCC patients, which exhibited phenotypic diversity and suggested potential functional specialization (Fig. 2a). The expression patterns of protein markers from our panel are shown in the corresponding dot plots (Fig. 2b). To evaluate their infiltration in various tissues, we quantified the frequencies of different CD4^+^ T-cell lineages in blood, adjacent nontumor tissues, and tumor tissues. In ESCC tumor tissues, Tregs (C20, C21, and C24) presented high infiltration rates, accounting for 48.5% of the immune cell population, which was significantly greater than that in blood (6.0%) and adjacent nontumor tissues (17.1%) (Fig. 2c, d). This observation reflects a prominent immunosuppressive signature within the tumor microenvironment, a feature common across multiple cancer types.^8,12,13^ Phenotypically, tumor Tregs were CD25^+^ FOXP3^+^ with ICOS coexpression, consistent with an activated regulatory state (Fig. 2b).

Within the Treg compartment, we identified a CD25^+^FOXP3^+^CTLA4^+^CD39^+^ subset (C20) that was significantly enriched in tumor relative to blood and adjacent nontumor tissue. A second subset, C21 (CD25^+^FOXP3^+^CTLA4^-^CD39^+^) likewise preferentially accumulated intratumorally. Furthermore, our investigations revealed that PD-1^+^ Treg cells (C24) are enriched in tumor tissues.

In the blood, the predominant CD4^+^ T cells, each representing more than 5.0% of the total, included a naïve type (C13), a central memory type (TCM) (C14, characterized by CD127 expression), and an effector-like, partially exhausted phenotype (C16, characterized by GZMB and T-bet expression^14,15^) (Fig. 2c). Notably, C13 and C16 were abundant in blood (C13, 33.6%; C16, 13.6%) but rare in adjacent nontumor tissues (C13, 0.2%; C16, 0.4%) and cancerous tissues (C13, 0.1%; C16, 0.3%) (Fig. 2d).

In ESCC tumor tissues, the median percentage of TCM (C12) was 16.7% in adjacent nontumor tissues and 6.7% in tumor tissues, with the lowest percentage observed in ESCC blood at 3.6% (Fig. 2d). We identified CD4^+^CCR4^+^CXCR3^+^ Th cells (Cluster C11), which were notably more prevalent in adjacent nontumor tissues (10.4%) than in tumor tissues (5.2%) and blood (0.9%). Additionally, we identified two populations of CD4^+^ tissue-resident memory (TRM) cells characterized by CCR5 expression.^23^ Specifically, Cluster C17 was enriched in adjacent nontumor tissues (28.9%), whereas Cluster C25 was predominant in tumor tissues (3.9%) (Fig. 2c, d).

### Characterizing CD8^+^ T-cell phenotypes and TCM antitumor strategies in the ESCC TME

We classified predefined CD8^+^ T cells into 11 distinct groups on the basis of their phenotypic markers. The classification included one naïve group (C09, CD45RA^+^CCR7^+^CD127^+^); two central memory (TCM) groups (C08/C10, CD45RA^−^CD127^+^/CD45RA^−^CCR7^+^); four TEX-like groups (C27, C28, C30, C34) that coexpressed canonical exhaustion markers (e.g., CD39, PD-1, TIM-3) and low T-bet levels, consistent with previous reports^14,15^; one effector memory (TEM) group (C38, CD45RA^-^CCR7^-^GZMB^+^); one terminally differentiated effector memory RA (TEMRA) group (C37, CD45RA^+^CCR7^-^); and two tissue-resident memory (TRM) groups (C31/32, CD45RA^−^CCR5^+^) (Fig. 2e, f).

Quantification of CD8⁺ T-cell lineages revealed pronounced heterogeneity with compartment-specific distributions (Fig. 2g, h). Among the CD8^+^ T-cell subgroups, TEX-like cells displayed significant heterogeneity. Specifically, CD39^+^PD-1^+^ TEX-like cells (C27/28) were more prevalent in tumor tissues (C27, 23.9%; C28, 7.1%), with C27 also exhibiting high expression of ICOS. CCR5^+^PD-1^int^ TEX-like cells (C30, 25.2%) were also predominantly found in tumor tissues, whereas CD38^+^TIM3^+^PD-1^+^ TEX-like cells (C34, 0.6%) were primarily detected in the blood (Fig. 2g, h). These findings indicate a tumor microenvironment enriched for exhausted T-cell states. In line with sustained antigenic stimulation in ESCC, these cells display blunted proliferative capacity and attenuated effector-cytokine production. However, these cells also retain the residual capacity to recognize and respond to tumor antigens, albeit at an impaired level. Compared with those in blood, CD8^+^ TCM cells were enriched in tumor and adjacent nontumor tissues (Fig. 2g, h). Within the CD8^+^ TCM population, C10 cells, which express the lymphoid homing marker CCR7, were more prevalent in adjacent nontumor tissues (14.8%), whereas CCR7^int^CD127^+^ C08 cells were more prevalent in the blood (6.1%). CD8^+^ naïve cells (C09, 17.3%) and TEMRA cells (C37, 63.8%) were detected primarily in the blood. Two TRM groups (C31/32) were significantly enriched in adjacent nontumor tissues (C31, 19.6%; C32, 37.8%) (Fig. 2g, h).

We investigated whether TCM supplementation could enhance antitumor cytotoxicity in vitro as an exploratory model to understand the functional relevance of these effects. We identified a critical deficiency of both CD4^+^ and CD8^+^ TCMs in the TME of ESCC tumors (Fig. 2g, h). To address this deficiency, we generated autologous ESCC patient-derived organoids (PDOs) from two patients and purified CD62L⁺ TCMs from matched peripheral blood mononuclear cells (PBMCs). We then established autologous, HLA-compatible PDO-TCM cocultures for 5 days, after which tumor-cell viability was quantified by flow cytometry in PDOs cultured with or without TCMs (Fig. 2i, Supplementary Fig. 3a, and Supplementary Table 4). This model elicited a sustained cytotoxic response, supporting the in vitro cytotoxic potential of CD62L⁺ TCM cells (Fig. 2j).

In parallel, TCM cells from four ESCC patients were cocultured with four ESCC cell lines (Supplementary Fig. 3b, c and Supplementary Table 5). This approach significantly increased antitumor efficacy, underscoring the pivotal role of TCM cells in reshaping the immune landscape and restraining tumor progression.

### PD-L1-high macrophages associate with immunotherapy benefit

**Fig. 3:**
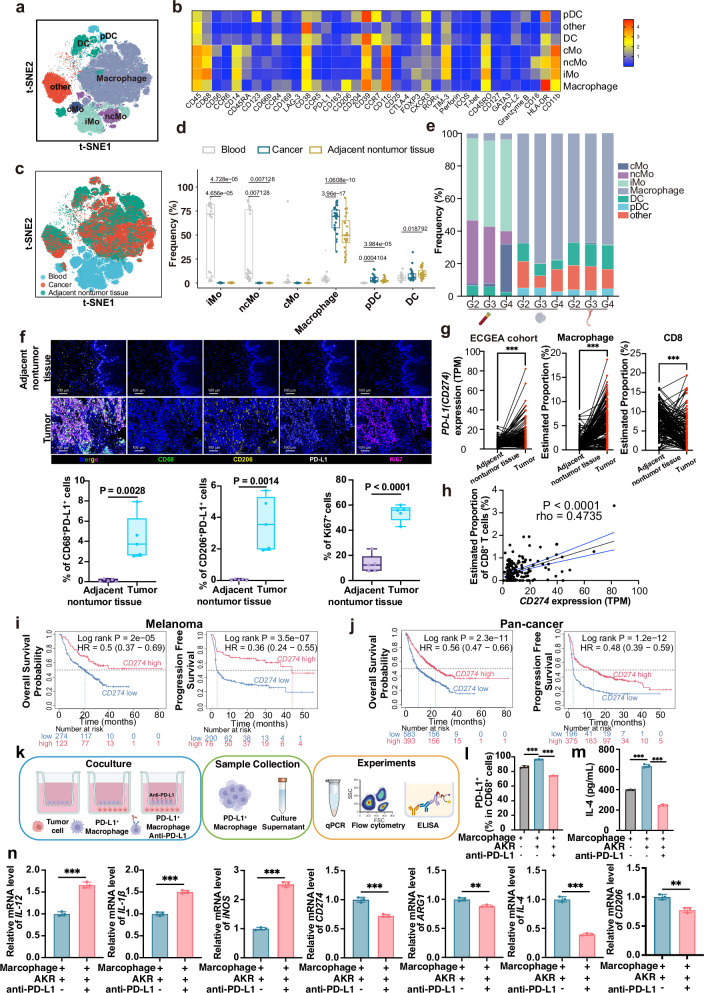
High PD-L1 expression in macrophages is correlated with improved immunotherapy outcomes. **a** t-SNE visualization of the distribution of myeloid cell subpopulations in ESCC patients, including classical monocytes (cMo), intermediate monocytes (iMo), nonclassical monocytes (ncMo), dendritic cells (DCs), plasmacytoid dendritic cells (pDCs), macrophages, and other myeloid cells. **b** Heatmap showing the average expression levels of representative protein markers in myeloid cell subpopulations. **c** t-SNE visualization of the origins of myeloid cell subpopulations in ESCC patients. **d** Box-and-whisker plots showing the distribution of myeloid cell subpopulations in patients with different ESCC origins. Kruskal–Wallis test with Benjamini–Hochberg post hoc correction. **e** Stacked bar plots presenting major myeloid cell frequencies in the blood, adjacent nontumor and tumor tissues of patients with ESCC. **f** mIHC to explore the spatial distribution of immune cells within ESCC tissues. mIHC with anti-CD206 (yellow), anti-CD68 (green), anti-Ki67 (magenta), and anti–PD-L1 (white) antibodies was used to count the immune cell types in the ESCC samples. Scale bars, 100 μm. **g** *CD274* (PD-L1) expression levels in ESCC tumors compared with adjacent nontumor tissues and the proportions of infiltrating macrophages and CD8^+^ T cells in ESCC tumors compared with adjacent nontumor tissues. Immune cell proportions were quantified via the CIBERSORT algorithm on the basis of bulk RNA-seq data from the ECGEA cohort. *P* < 0.001 (***), Wilcoxon signed-rank. **h** Correlation between *CD274* mRNA levels and CD8^+^ T-cell infiltration in ESCC tumor samples from the ECGEA cohort (RNA-seq). Spearman’s rank coefficient (rho) and two-sided P are reported. **i**, **j** Kaplan‒Meier curves for OS and PFS in melanoma (**i**) and pan-cancer (**j**) immunotherapy cohorts stratified by high versus low *CD274* expression (Kaplan‒Meier Plotter). Log rank *P* shown. **k** Diagram of macrophage and tumor cell coculture patterns. **l**–**n** Flow-cytometric quantification of PD-L1 (**l**) and ELISA of IL-4 in culture supernatants (**m**), one-way ANOVA test followed by post hoc test with Benjamini‒Hochberg correction; and qRT-PCR of *IL-12*, *IL-1β*, *iNOS*, *CD274*, *ARG1*, *IL-4* and *CD206* across conditions (**n**), two-tailed t test. Data are means ± SDs (*n* = 3). *P* < 0.01 (**), *P* < 0.001 (***). cMo classical monocytes, iMo intermediate monocytes, ncMo nonclassical monocytes, DCs dendritic cells, pDCs plasmacytoid dendritic cells

We delineated seven myeloid phenotypes within the ESCC tumor microenvironment: classical monocytes (CD14^+^CD16^-^), intermediate monocytes (CD14^+^CD16^int^), nonclassical monocytes (CD14^+^CD16^+^), plasmacytoid dendritic cells (pDCs; CD123^+^HLA-DR^+^CD14^-^CD16^-^), CD14^+^ dendritic cells (CD11c^+^CD14^+^), macrophages (CD206^+^HLA-DR^+^CD204^-^) and a residual other/rare category (Fig. 3a–c, Supplementary Fig. 4). Compositions varied by tissue, indicating context-specific remodeling of the myeloid compartment in ESCC.

We profiled peripheral-blood myeloid cells in ESCC and observed distinct monocyte distributions across clinical stages, consistent with progression-related remodeling of circulation-derived myeloid cells. Intermediate monocytes (50.3%) and nonclassical monocytes (32.4%) were enriched predominantly in the blood of ESCC patients, particularly during early-stage disease (grade information: G2 and G3), with less than 1% of these monocytes present in tumor and adjacent nontumor tissues. Conversely, classical monocytes comprised a smaller fraction (4.8%) of the blood overall but exceeded 30% in late-stage (grade information: G4) ESCC patients (Fig. 3d, e).

We observed distinct patterns of DC subpopulation distributions within tumor and adjacent nontumor tissues. DCs coexpressing CD14 and CD11c were enriched in both tumor (8.0%) and adjacent nontumor tissues (10.1%), with a notably greater prevalence in adjacent nontumor areas. In contrast, pDCs (CD123^+^HLA-DR^+^CD14^−^CD16^−^) were relatively increased in tumors (4.1% vs 2.6% in adjacent nontumor tissue) (Fig. 3b–d).

Compared with blood (3.6%), a CD206^+^HLA-DR^+^CD204^−^ macrophage subtype was predominantly enriched in tumor tissues (66.2%) and adjacent nontumor tissues (51.7%) (Fig. 3d). Using mIHC, we confirmed the high expression of these markers in ESCC tumor tissues, with frequent colocalization with PD-L1 (Fig. 3f). Analysis of ECGEA RNA-seq cohort^5^ showed high macrophages signatures in tumors than in adjacent nontumor tissues, accompanied by reduced CD8^+^ T cells and elevated *CD274* (PD-L1) expression (Fig. 3g).

Correlation analysis showed that *CD274* (PD-L1) expression was positively associated with intratumoral CD8^+^ T cells abundance (rho = 0.4735, *P* < 0.0001; Fig. 3h). This aligns with an “inflamed” phenotype in which PD-L1 marks pre-existing T-cell infiltration and greater likelihood of benefit from checkpoint blockade. In an external melanoma cohort, *CD274*^high^ exhibited improved survival: OS (HR = 0.50; 95% CI 0.37–0.69; log rank *P* = 2 × 10^−5^) and PFS (HR = 0.36; 95% CI 0.24–0.55; log rank *P* = 3.5 × 10^−7^) (Fig. 3i). Concordant results were seen across pan-cancer immunotherapy datasets: OS (HR = 0.56; 95% CI 0.47–0.66; log rank *P* = 2.3 × 10^−11^) and PFS (HR = 0.48; 95% CI 0.39–0.59; log rank *P* = 1.2 × 10^−12^) (Fig. 3j). Together, these data support PD-L1—including on myeloid cells—as a response correlate and a practical enrichment marker for ESCC immunotherapy studies. To test the functional plasticity of PD-L1^+^ macrophages, we isolated murine macrophages, induced PD-L1 via tumor coculture, and then treated them with an anti–PD-L1 antibody. At endpoint, cells and supernatants were analyzed by flow cytometry, ELISA and qRT-PCR (Fig. 3k). Flow cytometry revealed that tumor-induced macrophages upregulated PD-L1 expression, which was significantly reduced following anti–PD-L1 treatment (Fig. 3l). ELISA analysis revealed that the immunosuppressive cytokine IL-4 was markedly elevated after coculture, but its secretion was significantly diminished upon PD-L1 blockade (Fig. 3m). Transcriptionally, PD-L1 inhibition increased proinflammatory/antitumor genes (*IL-12*, *IL-1β* and *iNOS*), and downregulated immunosuppressive/protumor genes (*IL-4*, *ARG1*, *CD206* and *CD274*) (Fig. 3n). Collectively, these findings suggest that targeting PD-L1⁺ macrophages not only diminishes their immunosuppressive phenotype but also promotes functional reprogramming toward an antitumor inflammatory state.

### Intratumoral CD39⁺ T cells correlate with better prognosis

**Fig. 4:**
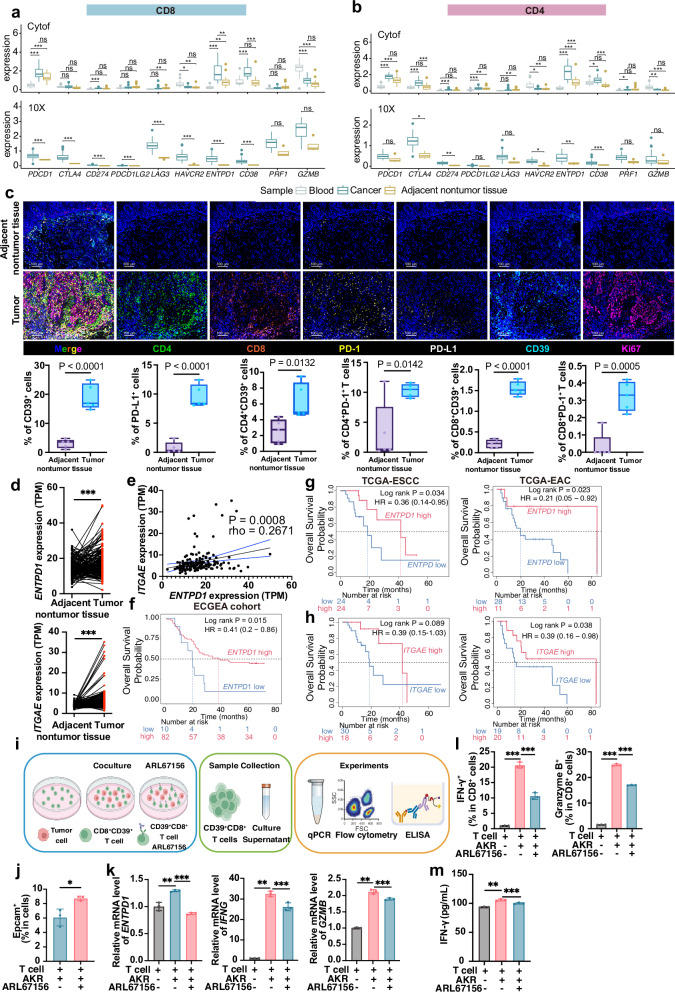
High CD39 on tumor-infiltrating T cells associates with favorable prognosis. **a**, **b** CyTOF and 10X Genomics analyses of functional marker expression in CD8^+^ T (**a**) and CD4^+^ T (**b**) cells in ESCC. Centre line, median; box, interquartile range; whiskers, 1.5× IQR. CyTOF, Kruskal–Wallis test with Benjamini–Hochberg post hoc correction; 10X Genomics, Mann‒Whitney *U* test. **c** mIHC showing spatial distribution of PD-1 (yellow), CD4 (green), Ki67 (magenta), CD39 (cyan), CD8 (red) and PD-L1 (white) in ESCC. Scale bars, 100 μm. **d** *ENTPD1* and *ITGAE* expression levels in ESCC tumors versus adjacent nontumor tissues (ECGEA RNA-seq). *P* < 0.001 (***), paired two-tailed t test. **e** Scatter plot showing the correlation between *ENTPD1* expression levels and *ITGAE* expression levels in ESCC tumor tissues, as assessed via RNA-seq data from the ECGEA cohort. Spearman’s rank coefficient (rho) and two-sided P are reported. **f** A Kaplan‒Meier survival analysis evaluating the impact of different *ENTPD1* expression levels within the context of high CD8^+^ T-cell infiltration in the ESCC ECGEA cohort. Log rank P indicated. **g**, **h** Kaplan‒Meier OS curves for esophageal squamous and adenocarcinoma cohorts (TCGA-ESCC/EAC) comparing high versus low *ENTPD1* (**g**) and *ITGAE* (**h**) within CD8^high^ tumors. Log rank *P* reported. **i** Diagram of T-cell and tumor cell coculture patterns. **j** Tumor cell viability after coculture with CD8⁺ T cells ± CD39 inhibitor (ARL67156). The data are presented as the means ± SDs (*n* = 3). Two-tailed t tests. *P* < 0.05 (*). **k**-**m** qRT-PCR analyses of *ENTPD1*, *IFNG* and *GZMB* levels; flow cytometry of PD-1, IFN-γ, and Granzyme B; and ELISA quantification of IFN-γ in culture supernatants. The data are presented as the means ± SDs; *n* = 3. One-way ANOVA test followed by post hoc test with Benjamini‒Hochberg correction. *P* < 0.01 (**), *P* < 0.001 (***)

Our comprehensive investigation commenced with CyTOF and 10X Genomics analyses, which revealed significantly elevated *ENTPD1* (CD39) expression in intratumoral CD8^+^ and CD4^+^T cells (Fig. 4a, b). This consistency between the single-cell RNA and protein levels of CD39 in ESCC was confirmed through subsequent validation via mIHC, highlighting the abundant CD39 on tumor-infiltrating CD8⁺ and CD4⁺ T cells (Fig. 4c).

In colorectal cancer, the coexpression of CD39 and CD103 delineates neoantigen-specific cytotoxic T cells (CTLs), particularly in tumors with low mutation burdens, indicating a functional antitumor immune response.^24^ In the ECGEA ESCC cohort, tumor tissue showed higher *ENTPD1* (CD39) and *ITGAE* (CD103) transcript abundance than adjacent nontumor tissue (Fig. 4d), and the two markers were positively correlated (rho = 0.2671, *P* = 0.0008; Fig. 4e). When stratified by CD8⁺ T-cell enrichment, *ENTPD1*^high^ tumors had superior survival (HR = 0.41, 95% CI 0.20–0.86, log rank *P* = 0.015; Fig. 4f). This trend was further validated in the esophageal carcinoma dataset from The Cancer Genome Atlas (TCGA-ESCA) –across both ESCC and EAC–where *ENTPD1* and *ITGAE* each linked to improved outcomes in CD8-high tumors (Fig. 4g, h). Comparable patterns were observed in cervical squamous cell carcinoma (TCGA-CESC) and head and neck squamous cell carcinoma (TCGA-HNSC) (Supplementary Fig. 5a, b),^25^ which were included because of their shared squamous histology with ESCC. These consistent observations across different datasets and cancer types strongly support the conclusion that CD39^+^ TILs serve as a robust marker of favorable prognosis.

To probe the function of CD39⁺ T cells within the TME, we isolated CD8⁺ T cells from mice, induced CD39 expression through coculture with tumor cells, and subsequently treated the cells with the CD39 inhibitor ARL67156. At endpoint, cells and supernatants were analyzed via qRT-PCR, flow cytometry, and ELISA (Fig. 4i). CD39 blockade diminished cytotoxicity, evidenced by an increase in viable tumor cells (Fig. 4j). Coculture markedly upregulated *ENTPD1* expression in CD8⁺ T cells, whereas ARL67156 was associated with reduced *ENTPD1* expression and attenuated effector programs, incuding lower *IFNG* and *GZMB* mRNA (Fig. 4k), fewer IFN-γ⁺ and GZMB⁺ CD8⁺ T cells by flow cytometry, and decreased secreted IFN-γ by ELISA (Fig. 4l, m). Together, these data indicate that CD39⁺ CD8⁺ T cells contribute to antitumor cytotoxic responses and that pharmacological CD39 inhibition compromises their effector function within the TME. The results emphasise the context-dependent role of CD39 in regulating T-cell activity and argue for precision targeting of CD39 on defined T-cell subsets rather than indiscriminate blockade.

### Patients with high expression of CD39 benefit from immunotherapy

**Fig. 5:**
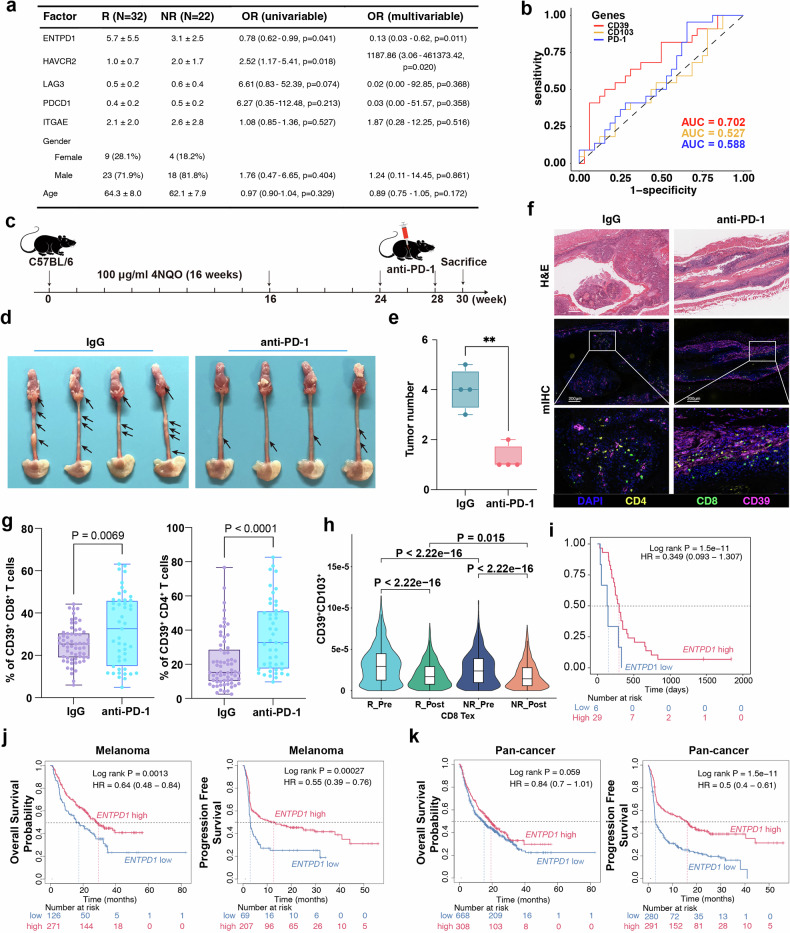
Patients with high expression of CD39 benefit from immunotherapy. **a** Univariate and multivariate Cox regression analyses assessing the association between immunotherapy response and T-cell exhaustion markers. **b** AUC for CD39, CD103 and PD-1 expression for the prediction of immunotherapy response. **c** Schematic diagram showing the 4NQO-induced ESCC mouse model. **d** Representative esophagus from 4NQO-induced ESCC mice with or without anti–PD-1 treatment; black arrows indicate 4NQO-induced tumor foci. **e** The number of tumors in the esophagus of 4NQO-induced ESCC mice treated with or without mouse anti–PD-1 therapy. *n* = 4, means ± SDs, unpaired t test, **P < 0.01. **f** mIHC staining of CD4 (yellow), CD8 (green), and CD39 (magenta) antibodies in esophageal sections from 4NQO-induced ESCC mice treated with or without mouse anti–PD-1. Scale bars, 200 μm. **g** CD39^+^CD8^+^ and CD39^+^CD4^+^ T cells among all nucleated cells per tissue core in 4NQO-induced ESCC mice treated with or without mouse anti–PD-1. **h** Violin plots of CD39 and CD103 in CD8⁺ exhausted T-cell clusters at the pretreatment (Pre) and posttreatment (Post) stages, stratified by clinical response (responders [R] vs. nonresponders [NR]). Kruskal–Wallis test with Benjamini–Hochberg post hoc correction. **i** Kaplan–Meier OS in the ECGEA cohort receiving anti–PD-1 monotherapy, stratified by high versus low *ENTPD1* expression; log rank *P* shown. **j**, **k** K‒M survival analysis comparing OS and PFS in melanoma (**j**) and pancancer (**k**) immunotherapy cohorts stratified by high versus low *ENTPD1* expression (Kaplan‒Meier plotter); log rank *P* reported

Our univariate Cox regression analysis of canonical T-cell exhaustion markers revealed that elevated *ENTPD1* (CD39) expression was significantly associated with improved outcomes under anti–PD-1 immunotherapy in ESCC patients^26^ (*P* = 0.041; Fig. 5a). In a multivariable model, CD39 remained an independent correlate of benefit (*P* = 0.011), consistent with a contributory role for CD39⁺ CD8⁺ T cells in antitumor immunity in ESCC. For prediction, *ENTPD1* (CD39) achieved an area under the ROC curve (AUC) of 0.702 with specificity 0.938 and a sensitivity 0.409 (Fig. 5b), outperforming both *ITGAE* (CD103) and *PDCD1* (PD-1). These findings support CD39 as a reliable biomarker for predicting responsiveness to PD-1 blockade in ESCC patients.

In the 4NQO-induced ESCC mouse model (Fig. 5c), anti–PD-1 therapy reduced esophageal tumor burden (Fig. 5d, e). Multiplex immunohistochemistry analyses of the mouse model revealed higher frequencies of CD39⁺CD8⁺ and CD39⁺CD4⁺ T cells in anti–PD-1-treated mice compared with IgG controls (Fig. 5f, g).

Re-analysis of the public scRNA-seq cohort from Liu et al.’s^27^ treated with immune checkpoint inhibitors. Stratification by clinical response revealed that, prior to treatment (Pre), CD39⁺ T cells were significantly enriched in responders (R-Pre) compared with nonresponders (NR-Pre). Following treatment (Post), the frequencies of both cell populations decreased overall but remained markedly greater in the responder group (R-Post vs. NR-Post) (Fig. 5h).

This pattern of T-cell enrichment was consistent across multiple patient cohorts who had undergone immunotherapy. We observed a consistent enrichment of CD39^+^CD8^+^ and CD39^+^CD4^+^ T cells in patients who received immune-based treatments. Further analysis of published data from melanoma (dbGaP: phs000452,^28^ ENA: PRJEB23709,^29^ GEO: GSE91061,^24^ and non-small cell lung cancer (NSCLC)^25^ (GEO: GSE126044), responders (R) to anti–PD-1 therapy harbored higher baseline frequencies of these CD39⁺ T-cell subsets than non-responders (NR) (Supplementary Fig. 5c). Notably, the posttreatment decline supports the dynamic modulation of these immune subsets during the checkpoint blockade. Together, these data indicate that elevated pretreatment CD39⁺ T cells associate with favorable immunotherapy outcomes and may serve as a practical enrichment biomarker, while their on-therapy contraction supports a functional role in mediating response.

Our ECGEA cohort of ESCC patients receiving anti–PD-1 monotherapy demonstrated that high *ENTPD1* (CD39) expression was associated with improved clinical outcomes (log rank *P* = 1.5 × 10^−^^11^), with a Cox HR of 0.35 (95% CI 0.09–1.31), indicating a favorable trend with imprecise effect (Fig. 5i). This observation is corroborated by data from melanoma studies, where high *ENTPD1* expression is also associated with considerable clinical benefits: OS (HR = 0.64; 95% CI 0.48–0.84; log rank *P* = 0.0013) and PFS (HR = 0.55; 95% CI 0.39–0.76; log rank *P* = 0.00027) (Fig. 5j). In pancancer immunotherapy cohorts, *ENTPD1*^high^ tumors similarly tracked with benefit: OS (HR = 0.84; 95% CI 0.7–1.01; log rank *P* = 0.059) and PFS (HR = 0.5; 95% CI 0.4–0.61; log rank *P* = 1.5 × 10^−^^11^) (Fig. 5k).

The observed increase in CD39^+^CD8^+^ and CD39^+^CD4^+^ T cells after PD-1 blockade points to a role for the CD39 axis in shaping treatment efficacy. Across models and patient cohorts, higher CD39⁺ T-cell abundance tracked with better clinical response, highlighting CD39 as a practical biomarker for response prediction and real-time monitoring under checkpoint inhibition. This cross-validation enhances the prospect of developing targeted therapies aimed at modulating CD39 activity to enhance antitumor immunity.

## Discussion

The comprehensive CyTOF analysis conducted on ESCC patients offers a nuanced view of the immune landscape, revealing a complex interplay of immune cell types and states. Compared with single-cell transcriptomic data, our study provides greater clarity on the distinct immune profiles in tumors, adjacent nontumor tissues, and peripheral blood samples, providing critical insights into the spatial and phenotypic heterogeneity of the immune response in ESCC. One of the pivotal findings from our analysis is the significant overlap in immune cell profiles among ESCC patients, suggesting potential commonalities in immune response mechanisms. This observation underscores the possibility of identifying universal therapeutic targets within the TME across different individuals suffering from ESCC. By contrast, peripheral blood displayed features not mirrored in tissue, underscoring its potential utility for prognosis and non-invasive immune monitoring. A salient finding was the enrichment of γδ T cells in node-positive patients (Fig. 1g). Similar correlations have been described in other malignancies: IL-17–producing γδ T cells recruit protumor neutrophils and foster metastatic spread in breast cancer,^30^ and promote angiogenesis with adverse outcomes in gallbladder cancer.^31^ These observations support a model in which subset-specific γδ T-cell programs remodel the TME via cytokines: enhancing neutrophil influx, angiogenesis, and suppression of cytotoxic immunity. Nevertheless, γδ T cells also possess potent antitumor properties, including direct cytotoxicity against tumor cells and antigen-independent immune surveillance. This functional dichotomy has been reviewed by Corsale et al. who highlighted the context-dependent protumor and antitumor effects of γδ T cells.^32^ Further phenotypic and functional dissection of γδ T-cell subsets in ESCC is warranted to delineate their precise roles in immune regulation and metastasis.

**Fig. 6:**
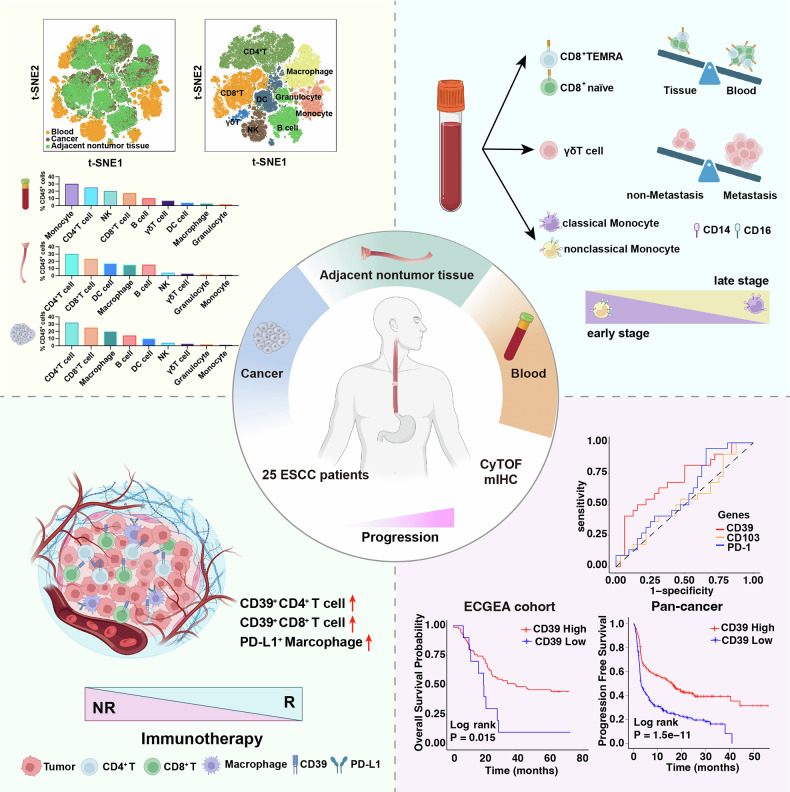
Summary of immune cell features in the blood and tissues of patients with ESCC. Schematic overview of the study and key findings in 25 patients with matched blood, adjacent nontumor tissue and tumor, profiled by CyTOF (42-marker panel) and multiplex immunohistochemistry (mIHC). Top left: t-SNE maps depicting major immune lineages. Left: stacked bars summarising compartmental frequencies across blood, adjacent tissue and tumor. Right: illustrative shifts with disease context—enrichment of CD8^+^ TEMRA and CD8^+^ naïve T cells in blood versus tissue, increased γδ T cells with nodal metastasis, and redistribution of classical/non-classical monocytes (CD14/CD16)—and a stage gradient (early→late). Bottom left: conceptual tumor immune microenvironment under immunotherapy; responders (R) vs. nonresponders (NR). Features associated with response are highlighted (↑ CD4^+^CD39^+^ T cells, ↑ CD8^+^CD39^+^ T cells, ↑ PD-L1^+^ macrophages). Bottom right: performance of CD39, CD103 and PD-1 for response prediction (ROC/AUC) and survival associations (Kaplan–Meier) in the ECGEA ESCC cohort and pan-cancer immunotherapy datasets. Schematic created with BioRender.com

The high prevalence and phenotypic heterogeneity of exhausted CD8⁺ T cells reflect profound adaptation of the immune system to chronic antigenic stimulation in ESCC. As summarized in Fig. 6, our study highlights CD39⁺ T cells as both prognostic indicators and functional mediators of antitumor immunity in ESCC. In univariate Cox analyses, higher CD39 associated with better outcome under anti–PD-1 therapy (*P* = 0.041; Fig. 5a), and this signal remained independent in multivariable models (*P* = 0.011). In predictive modeling, CD39 achieved an AUC of 0.702 (specificity 0.938, sensitivity 0.409), outperforming CD103 and PD-1 in this dataset—supporting its use for patient enrichment. Mechanistically, our CyTOF analysis localized CD39⁺PD-1⁺CD8⁺ T cells to the exhausted compartment of the tumor, particularly within the C27 cluster. While these cells exhibit features of functional exhaustion, their coexpression of activation markers suggests a transitional or “reinvigoratable” state. This observation aligns with emerging evidence in other cancer types, where exhausted T cells can be functionally restored following immune checkpoint blockade.^33^ Thus, CD39 may delineate tumor-reactive T cells that are functionally constrained yet amenable to therapeutic rescue. In patients responding to PD-1 blockade, intratumoral CD39⁺CD8⁺ and CD39⁺CD4⁺ T cells increased concurrently, supporting CD39 as a response predictive signature. Functional assays concordantly showed that tumor coculture upregulated CD39 in CD8⁺ T cells, whereas ARL67156 (CD39 inhibitor) attenuated effector programs—*IFNG* and *GZMB* transcripts decreased by qRT-PCR, the fractions of IFN-γ⁺ and GZMB⁺ CD8⁺ T cells fell by flow cytometry, and secreted IFN-γ declined by ELISA—indicating that CD39⁺ CD8⁺ T cells mediate cytotoxicity and that CD39 blockade compromises this function within the TME.

These prognostic and mechanistic signals align with prior reports: CD39⁺ T cells have been described as diagnostic/prognostic markers in ESCC^19^; intratumoral CD39⁺CD8⁺ T cells within tertiary lymphoid structures associate with better immunotherapy responses.^20^ Moreover, the coexpression of CD39 and CD103^21^—previously described by Duhen et al.—defines tumor-reactive cytotoxic T cells in multiple cancers, further reinforcing the conserved immunological significance of this subset across tumor types.

However, the role of CD39 in cancer is highly context dependent. While higher CD39 associates with favorable prognosis in lung adenocarcinoma,^34^ esophageal adenocarcinoma,^35^ colorectal cancer,^36^ ovarian cancer,^37^ and HNSC,^21^ it has been linked to worse outcomes in the bladder,^38^ breast,^39^ gastric,^40^ and hepatocellular^41^ cancers. These divergent findings likely reflect the dual immunomodulatory role of CD39, which not only marks exhausted or tumor-reactive T cells but also contributes to adenosine-mediated immunosuppression, particularly when expressed on regulatory T cells and myeloid populations.^42,43^

Thus, CD39 serves as a dual-edged biomarker—both a readout of immune dysfunction/tumor reactivity and a therapeutic node. Our data support a model in which CD39⁺ T cells constitute a poised effector population that is capable of cytotoxicity but susceptible to exhaustion in the immunosuppressive TME. Therapeutically, modulating adenosinergic signalling to relieve suppression—while avoiding indiscriminate inhibition of CD39 on tumor-reactive T cells—may favour T-cell reinvigoration and augment responses to PD-1 blockade.

In vitro supplementation with TCM addressed a key deficiency observed in the ESCC TME, suggesting that these cells may possess functional attributes relevant for immunotherapy. These findings suggest that restoring TCM warrants further investigation as a potential approach to enhance antitumor responses, although its clinical relevance remains to be validated. The reintroduction of TCM cells into the ESCC tumor microenvironment specifically addresses the critical deficiency of functional memory T cells. This strategy not only replenishes a key immune component but also leverages the intrinsic properties of TCM cells, including long-term persistence and rapid antigen recall. In our coculture assays, autologous CD62L⁺ TCM cells exhibited robust cytotoxicity against PDOs, highlighting their capacity to mediate antitumor responses in vitro. However, these findings are limited to controlled in vitro conditions and do not fully recapitulate the immunosuppressive complexity of the in vivo TME. Although the observed cytotoxic activity supports functional TILs, more physiologically relevant models and clinical validation are warranted to determine the true translational potential of this approach. The multifaceted influence of CD8⁺ TCM cells on tumor progression likely involves not only direct cytotoxic effects but also indirect modulation of the immune landscape. These cells are associated with reduced tumor growth and improved survival in various cancer models, including those of colorectal cancer and melanoma.^44^ Future clinical trials are essential for evaluating the feasibility and efficacy of TCM-based strategies, potentially in combination with immune checkpoint inhibitors or other immunotherapies.

The distinct distribution and phenotypic specialization of myeloid compartment—spanning dendritic cells and monocyte lineages—emerges as a key determinant of immune tone in ESCC. Notably, CD16^+^ intermediateand non-classical monocytes, which exhibit heightened antigen-presenting capacity and proinflammatory cytokine output (TNF-α, IL-6), were preferentially enriched in early-stage disease.^45^ The enrichment of CD16^+^ monocytes in early-stage ESCC patients could indicate an initial robust immune response, potentially offering opportunities for therapeutic intervention aimed at enhancing monocyte-mediated immunity. This shift in distribution toward classical monocytes in later stages may reflect a change in the immune environment or disease progression mechanisms. These dynamics clarify how myeloid specialisation shapes the ESCC TME and point to stage-specific interventions—for example, amplifying CD16^+^ monocyte programs early versus reprogramming suppressive myeloid states later—to optimise immunotherapeutic responsiveness.

The substantial enrichment of CD206^+^CD204^−^HLA-DR^+^ macrophages in tumor and adjacent nontumor tissues, coupled with high PD-L1 expression, indicates a potential immune evasion mechanism that could be strategically targeted in immunotherapy. The covariation of macrophage abundance, PD-L1 levels, and CD8⁺ T-cell infiltration suggests a nuanced regulatory network within the TME that influences immunotherapy outcomes.

Our findings support the hypothesis that macrophage PD-L1 is not merely a passive marker but also actively contributes to the immunosuppressive landscape, thereby affecting the efficacy of PD-1/PD-L1–targeted therapies. The correlation of high PD-L1 expression with improved survival outcomes across various cancers, as evidenced by significant benefits in overall and progression-free survival, underscores its value as a potential universal biomarker for predicting immunotherapy response.

Recent studies have begun to uncover the functional complexity of PD-L1⁺ TAMs, challenging the traditional view of PD-L1 as a purely immunosuppressive marker. Wang *et al*. showed that PD-L1-expressing macrophages can exert immunostimulatory effects on breast cancer and are associated with favorable clinical outcomes.^16^ This observation parallels our finding that PD-L1⁺ macrophages are enriched in ESCC tumors, suggesting a more nuanced role in modulating immune responses. Importantly, we showed that PD-L1 blockade induces a transcriptional shift in TAMs, characterized by the upregulation of proinflammatory and antitumor genes (*IL-12*, *IL-1B*, and *iNOS*) and the downregulation of immunosuppressive and protumor genes (*IL-4*, *ARG1*, *CD206* and *CD274*) (Fig. 3n). These findings indicate that targeting PD-L1⁺ macrophages may not only reduce immunosuppression but also reprogram TAMs toward a proinflammatory, antitumor phenotype. Additional studies, such as those by Ma et al. and Elomaa et al., emphasized the context-dependent immunoregulatory functions of PD-L1⁺ macrophages, which may vary between cancer types and immune microenvironments.^17,18^ These insights highlight the importance of further dissecting PD-L1⁺ myeloid subpopulations in ESCC patients to better understand their prognostic and therapeutic significance and to refine their use as predictive biomarkers for immunotherapy.

Future work should focus on dissecting the functional contributions of different myeloid cell subsets to PD-L1-mediated immune regulation. This understanding will be crucial for developing targeted therapies that not only increase the efficacy of existing immunotherapy regimens but also extend the benefits to a broader cohort of ESCC patients, potentially transforming the therapeutic landscape for this challenging malignancy.

## Materials And Methods

### Human ESCC tissue samples

This study was approved by the institutional review board and independent ethics committee of the National Cancer Center/Cancer Hospital, Chinese Academy of Medical Sciences (approval number: NCC2019C-20), and complied with the Declaration of Helsinki and the International Council for Harmonisation Good Clinical Practice (ICH-GCP). Written informed consent for tissue procurement and study participation was obtained from all participants. We prospectively enrolled 25 consecutive, treatment-naïve patients with histologically confirmed ESCC between May 2019 and July 2020 at the Cancer Hospital, Chinese Academy of Medical Sciences (Supplementary Table 1). Tumor histology was assigned according to World Health Organization criteria, and clinical stage was determined using the UICC/AJCC TNM staging system (8th edition).

### Tissue preparation

Immediately after surgical resection, fresh specimens were placed into chilled preservation buffer (Miltenyi Biotec) and maintained at 4 °C during transport; processing was completed within 24 h. Tissues were mechanically minced with sterile scalpels and enzymatically dissociated using a commercial tumor dissociation kit on a gentleMACS Dissociator (both Miltenyi Biotec). Resulting single-cell suspensions were sequentially passed through 70 µm and 40 µm sterile strainers. Cell viability was assessed by a 1 min 25 µM cisplatin pulse (Enzo Life Sciences) followed by quenching in serum-containing buffer. Cells were then fixed in 1.6% paraformaldehyde for 10 min at room temperature (Electron Microscopy Sciences) and stored at −80 °C.

### Mass-tag cellular barcoding

Immune profiling used a 42-marker CyTOF panel on matched compartments. Acquisition, bead normalization, debarcoding, spillover compensation and gating followed established workflows. Clustering and visualization employed FlowSOM and t-SNE/UMAP. Detailed barcoding chemistry, panel composition, instrument settings and gating trees are provided in Supplementary Methods and Supplementary Table 2.

### Antibody conjugation and validation

Carrier-free antibodies were metal-conjugated and titrated on PBMCs and tumor-derived cells; specificity was verified by expected hierarchies and omission controls. Full clone lists, vendors, metal tags and catalogue numbers are provided in Supplementary Table 2 and Supplementary Methods. As an initial condition, antibodies were used at 1 µL per 3 × 10⁶ live cells in 100 µL staining buffer; all reagents were then re-titrated under experimental conditions to define optimal working concentrations. Reagent inventory and versioning were managed via the AirLab cloud platform.

### Antibody staining and cell volume quantification

Cells were Fc-blocked and stained with the immune panel; nuclei were labelled with iridium intercalator prior to Helios acquisition. Volume estimation used a ruthenium-based dye. Exact buffers, temperatures, incubation times and wash steps are provided in Supplementary Methods.

### CyTOF acquisition, preprocessing and unsupervised clustering

The CD45⁺ live singlet population was analyzed by unsupervised FlowSOM clustering on arcsinh-transformed intensities (cofactor=5); t-SNE/UMAP was used for visualization. Sequential gates (DNA⁺/live/CD45⁺/singlets, bead exclusion) defined high-quality events. Full gating templates, parameters and software versions are provided in Supplementary Methods.

### Multilabel immunohistochemistry staining

FFPE sections underwent heat-induced epitope retrieval and iterative multiplex staining for lymphoid/myeloid markers with DAPI counterstain. Image acquisition and quantification used standardised settings. Antibody panels, retrieval conditions and analysis pipelines are detailed in Supplementary Methods.

### 4NQO-induced ESCC mouse model

Male C57BL/6 J mice (6 weeks old) were exposed to 4-nitroquinoline-1-oxide (4NQO; 100 µg mL^−^¹) in the drinking water for 16 weeks to induce esophageal squamous carcinogenesis. After the exposure phase, animals received plain water until experimental endpoints. Mice were randomised to experimental groups prior to interventions. All procedures were approved by the Institutional Animal Care and Use Committee of the National Cancer Center/Cancer Hospital, Chinese Academy of Medical Sciences (approval number: NCC2019A106), and were performed in accordance with institutional guidelines.

### Mouse immune-cell isolation and functional assays

Murine CD8⁺ T cells were purified (negative selection) and activated with IL-2/anti-CD3 to induce CD39; PD-L1⁺ macrophages were generated from bone marrow using M-CSF with IL-4 polarisation. Macrophage and tumor interactions were tested in Transwell cocultures with or without anti–PD-L1. Endpoints included flow cytometry, qRT-PCR and ELISA for activation/polarisation markers and cytokines. Full protocols are provided in Supplementary Methods.

### RNA isolation and qRT-PCR

Gene expression was quantified by SYBR-based qRT–PCR using GAPDH as internal control. Primer sequences, cycling conditions and QC thresholds are listed in Supplementary Table 3 and Supplementary Methods.

### ELISA for determining IFN-γ and IL-4 concentrations

The IFN-γ concentration was determined via an ELISA Kit according to the manufacturer’s instructions (Elabscience Biotechnology, E-EL-M0048). The IL-4 concentration was determined via ELISA according to the manufacturer’s instructions (Elabscience Biotechnology, E-EL-M0043).

### ESCC PDO platform and autologous TCM-PDO cytotoxicity experiment

Autologous ESCC PDOs were established and cocultured with purified CD62L⁺ central memory T cells at defined effector-to-target ratios. Assay readouts included Annexin V/7-AAD cytotoxicity and cytokine measurements. Media composition, seeding density, timing, and analysis criteria are provided in Supplementary Methods.

### Statistical analysis

Correlations between gene expression and immune cell subsets were estimated via Pearson correlation. Survival rates were determined via the Kaplan‒Meier method. Statistical significance was assessed via the Kruskal‒Wallis test, one-way ANOVA, Mann‒Whitney *U* test, and t-test, with adjustments for multiple comparisons via the Benjamini‒Hochberg method.

## Supplementary information

Supplementary materials for Single Cell Atlas of Esophageal Squamous Cell Carcinoma Immune Ecosystem to Predict Immunotherapy Response

## Data Availability

Raw and processed mass cytometry data (.fcs files and metadata) from 25 ESCC patients have been deposited in the National Genomics Data Center (NGDC) OMIX repository under accession number OMIX011914. TCGA cohorts (TCGA-ESCA, TCGA-HNSC, TCGA-CESC) were accessed via UCSC Xena platform (https://xena.ucsc.edu/); cases lacking survival or RNA-seq data were excluded. For the ECGEA cohort, RNA-seq data, along with associated clinical annotations, are available at the GSA-Human HRA003107 (https://ngdc.cncb.ac.cn/gsa-human/browse/HRA003107). Public melanoma datasets were retrieved from GEO (GSE91061), ENA (PRJEB23709), and dbGaP (phs000452); the NSCLC dataset was from GEO (GSE126044). Survival and immunotherapy response data from the melanoma and pancancer immunotherapy cohorts were downloaded from the KM plotter immunotherapy database. All remaining data supporting the findings are available within the Article and its Supplementary Information. Source data are provided with this paper.
