# Supplementary material for: Single-cell atlas of the esophageal squamous cell carcinoma immune ecosystem to predict immunotherapy response
Source: Signal Transduct Target Ther. 2025 Oct 20;10:348. doi: 10.1038/s41392-025-02446-x (PMC12536040; doi:10.1038/s41392-025-02446-x)
Supplement: Supplementary file 1 — Supplementary materials for Single Cell Atlas of Esophageal Squamous Cell Carcinoma Immune Ecosystem to Predict Immunotherapy Response [file 41392_2025_2446_MOESM1_ESM.docx]

Supplementary Materials for

**Single-cell atlas of the esophageal squamous cell carcinoma immune ecosystem to predict immunotherapy response**

Xiankai Chen^#1^, Yahui Zhao^#2^, Yuhao Wang^#2^, Xiliang Wang^2^, Yuhao Liu^2^, Zhihua Liu*^2,3^, Yin Li*^1^

Correspondence to: liuzh@cicams.ac.cn, liyin_thorax@163.com

**This PDF file includes:**

Materials and Methods

Supplementary Text

Figures. S1 to S5

Tables S1 to S4

Materials and Methods

**Mass-tag cellular barcoding**

To control inter-sample staining variability, fixed cell suspensions were barcoded with metal mass tags^1^. We implemented a 126-well scheme based on unique 4-of-9 combinations. The barcode library comprised palladium isotopes (¹⁰⁵Pd, ¹⁰⁶Pd, ¹⁰⁸Pd, ¹¹⁰Pd; Fluidigm) coupled to bromoacetamidobenzyl-EDTA (Dojindo), together with indium (¹¹³In, ¹¹⁵In; Fluidigm), yttrium, rhodium and bismuth (⁸⁹Y, ¹⁰³Rh, ²⁰⁹Bi; Sigma-Aldrich) conjugated to maleimido-monoamide-DOTA (Macrocyclics). Working concentrations were: ²⁰⁹Bi, 20 nM; ¹⁰⁵^-^¹¹⁰Pd, ¹¹⁵In and ⁸⁹Y, 100 nM; ¹¹³In, 200 nM; and ¹⁰³Rh, 2 mM. Cells were randomized across two 96-well plates (~3 × 10^5^ cells per well) and barcoded under a transient partial permeabilization protocol. Prior to labeling, cells were rinsed once in PBS containing 0.03% saponin (Sigma-Aldrich), then incubated with barcoding reagent (200 µl per well) for 30 min at room temperature. Excess tag was removed by four washes in cell staining medium (CSM; PBS with 0.3% saponin and 0.5% BSA; Sigma-Aldrich) supplemented with 2 mM EDTA (StemCell Technologies). Wells were then pooled for downstream antibody staining. Two 126-well barcoding plates—each including an identical set of standard control samples—were subsequently stained with tumor-centric and immune-centric panels.

**Antibody conjugation and validation**

All antibodies (clones, vendors, metal isotopes) are listed in Supplementary Table 2. Specificity was confirmed in-house by titration on positive/negative controls, with isotype/background and competition (unlabeled antibody/peptide) where applicable. Antibodies were supplied carrier/protein-free or purified via Magne Protein A/G (Promega) before conjugation. Metal labeling used the Maxpar X8 Multimetal Kit (Fluidigm); reagents were cleaned up, buffer-exchanged, and retitrated to set working concentrations.

Each conjugated antibody was titrated and validated prior to use. Validation was performed using fresh human PBMCs stained with the metal-tagged antibody in parallel with appropriate negative controls (e.g., PBS). The antibody was conjugated with metal and validated by comparing the population with unstained controls. This ensured specificity and an appropriate signal-to-noise ratio. The recommended starting dilution for most antibodies is 1 μL per 3 × 10⁶ live cells in 100 μL of staining buffer. However, all the antibodies were titrated under experimental conditions to determine the optimal working concentration, and the antibodies were managed via the cloud-based platform AirLab as previously described^2^.

**Antibody staining and cell volume quantification**

Following mass-tag cellular barcoding, pooled cells were Fc-blocked (FcR Blocking Reagent, Miltenyi Biotec; 10 min, 4 °C) and washed in CSM. Samples were stained with the immune-focused primary panel (45 min, 4 °C), washed three times and—where applicable—incubated with a secondary goat anti-rabbit IgG conjugated to 165Ho (Vector Labs). For mass cytometry, the cells were incubated with a 500 nM iridium intercalator (¹⁹¹Ir/¹⁹³Ir; Fluidigm) in PBS containing 1.6% PFA (Electron Microscopy Sciences), washed, and then stained with a ruthenium complex for volume measurement^3^. The prepared cells were diluted in ddH2O with calibration beads, filtered, and analyzed on a Helios CyTOF2 instrument (Fluidigm), which captured the data in the .fcs format.

**CyTOF data acquisition**

Mass cytometry data were acquired on a Helios™ mass cytometer (Fluidigm). Cell events were bead-normalized via EQ™ Four Element Calibration Beads (Fluidigm) and analyzed via Cytobank and FlowJo software. To ensure high-quality data, a stringent gating strategy was applied:

**DNA⁺ cell selection:** Cells were first gated on the basis of DNA content via the ^191^Ir and ^193^Ir channels (^191^Ir/^193^Ir ). Events with low DNA signals, such as debris or doublets with asymmetric DNA distributions, were excluded.

**Live cell gating:** Viable cells were selected by excluding cisplatin-positive (^194^Pt⁺) events, which indicate membrane-compromised (dead) cells. A low signal in the ^194^Pt channel corresponds to live cells.

**Immune cell gating:** Immune cells were identified via CD45 (^89^Y), and further analysis was performed on the CD45⁺ population.

**Singlet selection:** Singlet cells were gated using event length versus DNA signal. Events with event lengths > 20 were retained to eliminate potential doublets or aggregates.

**Bead exclusion:** Sample acquisition beads were excluded on the basis of the ^140^Ce signal. Events with a high ^140^Ce intensity were considered beads or bead aggregates and were removed.

The cleaned CD45⁺ singlet live-cell population was used for downstream clustering and visualization, including t-SNE and FlowSOM analyses.

**Preprocessing of mass cytometry data**

The mass cytometry data were processed through a series of steps to ensure quality and accuracy. First, files were concatenated via the .fcs File Concatenation Tool (Cytobank, Inc.) and then normalized via the MATLAB version of the normalization tool^4^. Data debarcoding was performed via the CATALYST R/Bioconductor package^5^, followed by compensation for channel crosstalk via single-stained polystyrene beads. The adjusted .fcs files were uploaded to the Cytobank server for manual gating, with a focus on excluding nonspecific background and cisplatin-positive dead cells. Populations of interest were then exported and analyzed in R for further data validation and comparison between barcoding plates, increasing the consistency of the results presented.

**Dimensionality reduction and clustering**

Dimensionality reduction of mass cytometry data was performed via the t-SNE algorithm, as described by van der Maaten and Hinton^6^. The signal intensities per channel were transformed via an arcsinh function with a cofactor of 5 (counts_transf = asinh(x/5)). Notably, the same principal components were employed for nonlinear dimension reduction to facilitate the generation of visual projections via uniform manifold approximation and projection (UMAP) or t-distributed stochastic neighbor embedding (t-SNE). Specifically, t-SNE was applied for analyses of the entire tumor microenvironment. UMAP was employed for analyses focused on specific immune subsets, such as T cells.

**Multilabel immunohistochemistry staining**

Formalin-fixed tissue was processed into 5 µm paraffin sections and subjected to heat-induced antigen retrieval before immunostaining. Primary antibodies were applied against CD68 (1:200; Abcam, ab955/ab213363), CD206 (1:200; Abcam, ab64693), CD39 (1:100; Abcam, ab223842), CD169 (1:200; Abcam, ab227672), CD4 (1:200; Abcam, ab288724), CD8 (1:200; Abcam, ab217344/ab237709), PD-1 (1:100; Cell Signaling Technology, 84651), PD-L1 (1:100; Cell Signaling Technology, 64988) and Ki67 (1:800; Abcam, ab15580). After washes, sections were incubated with fluorophore-conjugated secondary antibodies—FITC goat anti-rabbit IgG (H+L) (1:1000; Proteintech, SA00003-2), TRITC goat anti-rat IgG (H+L) (1:1000; Proteintech, SA00007-7) and Alexa Fluor 647 goat anti-mouse IgG (H+L) (1:1000; Cell Signaling Technology, 4410)—followed by nuclear counterstaining with DAPI (Thermo Scientific, 62247). For multiplex detection, staining was performed using the Opal 7-Color Manual IHC Kit (Akoya Biosciences, NEL811001KT) according to the manufacturer’s instructions.

**Cell culture**

Human ESCC cell lines KYSE30, KYSE150, KYSE450, and KYSE510 were obtained from Dr. Shimada Y^7^. Cells were maintained in RPMI-1640 (HyClone) supplemented with 10% fetal bovine serum (HyClone) at 37 °C in a humidified 5% CO₂ incubator. Unless otherwise specified, cultures were expanded at low passage and passaged at ~70–80% confluence. Mycoplasma status was routinely monitored prior to experimental use.

**Cytotoxicity experiment**

Each group of TCM cells was uniformly mixed with ESCC cell lines and placed in a U-shaped 96-well plate, and the reaction volume was set to 200 μL. Three to four parallel duplicate wells were set; the effective target ratio (effector cells: target cells) were 16:1, and 32:1. 10,000 labeled target cells were added to each well, and then effector cells were added and centrifuged at 120 × g for two minutes and incubated for four hours in a cell culture incubator. After centrifugation at 120 × g for four minutes at 4 °C, the supernatant was discarded, and each well was resuspended in 200 μL of staining buffer and centrifuged twice. 125 μL staining buffer was added to each well and resuspended. The cell suspension was aspirated into a flow tube, to which 5 μL of Annexin V and 5 μL of 7-AAD was added. The reaction was performed for five minutes on ice, followed by analysis using flow cytometry.

**CD8^+^CD39^+^ T-cell isolation and induction**

CD8^+^ T cells were isolated via a CD8a^+^ T-cell isolation kit according to the manufacturer’s instructions (Miltenyi Biotec, 130--104--075). Well-coated plates were inoculated with 1640 medium containing 50 µM 2-mercaptoethanol. Mouse IL-2 was added to a final concentration of 10 ng/mL, and the mixture was activated for 48 h. T-cell medium containing 2 µg/mL CD3 was subsequently added and changed every 48 h, and CD8^+^CD39^+^ T cells were obtained after 96 h.

**PD-L1^+^ macrophage isolation and induction**

The mice were euthanized and subsequently surface sterilized by immersion in 75% alcohol. The femur and tibia were stripped with ophthalmic forceps, and the bone was cut from the joint and immersed in prepared basal medium. After the basal medium was aspirated with a 1 mL syringe, the needle was introduced from one end of the bone, and the marrow was removed, which was repeated 3 times until the bone turned white. The filtrate was centrifuged at 300 × g for 5 min and resuspended in 5 mL of erythrocyte lysate, after which the erythrocytes were lysed at 4 °C for 10 min. The reaction was terminated by the addition of an equal volume of complete medium. The cells were subsequently centrifuged at 300 × g for 5 min, after which the supernatant was discarded. The cells were resuspended in 30 ng/ml M-CSF complete medium at 37 °C in a humidified CO_2_ incubator. The medium was changed at 72 h, and every 48 h thereafter. Bone marrow-derived macrophages (BMDMs) were considered mature on day 7. To induce PD-L1, BMDMs were polarized with IL-4 (20 ng/mL) for 48 h.

**Macrophage and tumor cell coculture assay**

BMDMs were cocultured with AKR tumor cells at a 1:1 ratio in Transwell plates (0.4 μm pore size) for another 48 h. To assess the role of PD-L1, an anti–PD-L1 antibody (10 μg/mL) was added during the final 24 h of coculture. At endpoint, macrophages and supernatants were harvested for flow cytometry to evaluate PD-L1 expression, quantitative real-time PCR (qRT-PCR) to assess *CD274* and polarization markers, and ELISA to quantify cytokine secretion.

**RNA isolation and qRT-PCR**

Total RNA was isolated from cultured cells via TRIzol reagent (Thermo Fisher Scientific). Complementary DNA was generated via the Quantscript RT Kit (Tiangen, KR103) following the manufacturer’s protocol. Quantitative PCR was performed via PowerUp™ SYBR™ Green Master Mix (Applied Biosystems, A25742) on a StepOnePlus™ Real-Time PCR System (Applied Biosystems). Relative transcript abundance was calculated by the ΔΔCt method with GAPDH as the internal control. Primer sequences are listed in Supplementary Table 3.

**ESCC PDO platform and autologous TCM-PDO cytotoxicity experiment**

Fresh ESCC tissue was minced, enzymatically digested (collagenase IV/dispase II/DNase I; 30–60 min, 37 °C), filtered (70 µm) and embedded as 20–40 µl domes of growth-factor-reduced Matrigel. Domes were overlaid with Advanced DMEM/F12-based ESCC organoid medium containing essential supplements; Y-27632 (10 µM) was included for 24–48 h. Medium was changed every 2–3 days. Organoids were passaged every 7–10 days (1:3–1:4) by gentle dissociation and re-embedding.

For autologous killing assays, PDOs were dissociated into single cells using TrypLE Express (Thermo Fisher Scientific) and filtered through a 40 μm cell strainer. For co-culture experiments, PDO cells were seeded in 96-well U-bottom plates and incubated with autologous tumor-conditioned T cells (TCM) for 5 days. After co-culture, cells were harvested, washed with PBS, and stained with the following antibodies: anti-CD45 (PE-Cy7) to distinguish immune versus tumor cells, and Annexin V-FITC (BD Biosciences) in Annexin V binding buffer (10 mM HEPES, 140 mM NaCl, 2.5 mM CaCl₂; pH 7.4) to detect apoptotic/dead tumor cells. Following gating on live single cells based on FSC-A/SSC-A profiles, CD45⁻ tumor cells were selected, and Annexin V⁺ events were quantified as dead cells. Data were analyzed using FlowJo software. The proportion of Annexin V⁺ cells was used as a readout for T cell–mediated cytotoxicity. For samples containing both tumor cells and TCM cells, photomultiplier tube (PMT) voltages were adjusted to accommodate the differing size and granularity profiles of the two cell types, which resulted in a shift in forward and side scatter (FSC/SSC) parameters. To ensure accurate and biologically relevant gating, populations were defined based on relative distribution patterns rather than fixed coordinate values. Dead cells were identified using Annexin V staining within the CD45⁻ tumor cell population. The gating strategy was applied consistently across experimental conditions with respect to population identity, though minor adjustments were made to reflect differences in scatter profiles. To improve visualization and comparability, all flow cytometry plots were generated using a biexponential (Biex) scale on both axes. Additionally, pseudocolor plots were replaced with contour plots to enhance resolution and interpretability of gating boundaries.

Supplementary Text

**References**

1. Zunder, E. R. *et al.* Palladium-based mass tag cell barcoding with a doublet-filtering scheme and single-cell deconvolution algorithm. *Nat Protoc* **10**, 316-333 (2015).

2. Catena, R., Ozcan, A., Jacobs, A., Chevrier, S. & Bodenmiller, B. AirLab: a cloud-based platform to manage and share antibody-based single-cell research. *Genome Biol* **17**, 142 (2016).

3. Behbehani, G. K. *et al.* Transient partial permeabilization with saponin enables cellular barcoding prior to surface marker staining. *Cytometry A* **85**, 1011-1019 (2014).

4. Finck, R. *et al.* Normalization of mass cytometry data with bead standards. *Cytometry A* **83**, 483-494 (2013).

5. Chevrier, S. *et al.* Compensation of Signal Spillover in Suspension and Imaging Mass Cytometry. *Cell Syst* **6**, 612-620 e615 (2018).

6. Van der Maaten, L. & Hinton, G. Visualizing data using t-SNE. *J Mach Learn Res* **9**, 2579–2605 (2008).

7. Shimada, Y., Imamura, M., Wagata, T., Yamaguchi, N. & Tobe, T. Characterization of 21 newly established esophageal cancer cell lines. *Cancer* **69**, 277-284 (1992).

**Figure. S1.**

**
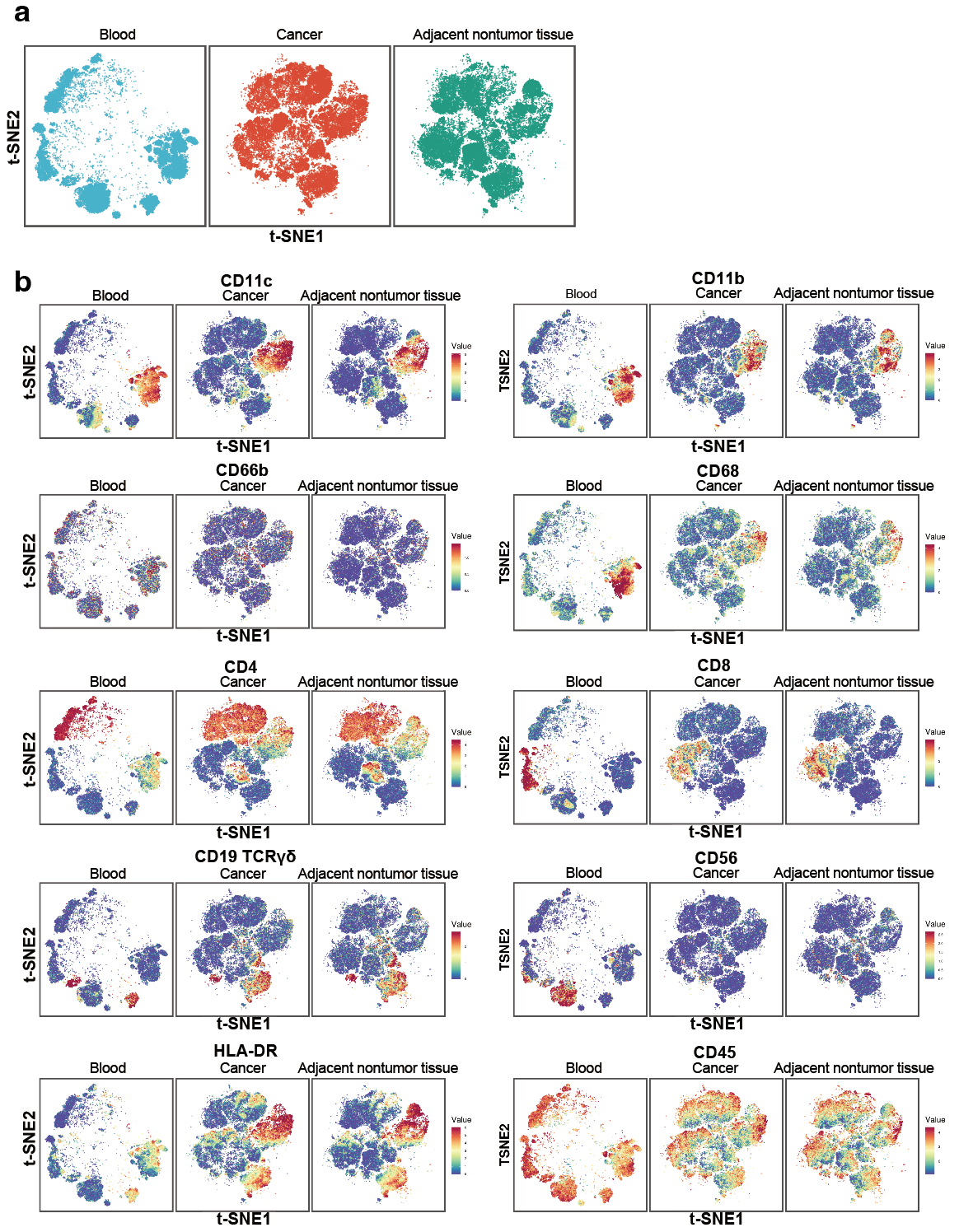
**

**Figure. S1.** **Major immune cell identification.**

1. t-SNE map showing the marker expression pattern in major immune cells.
2. Total immune subclass cell identified.


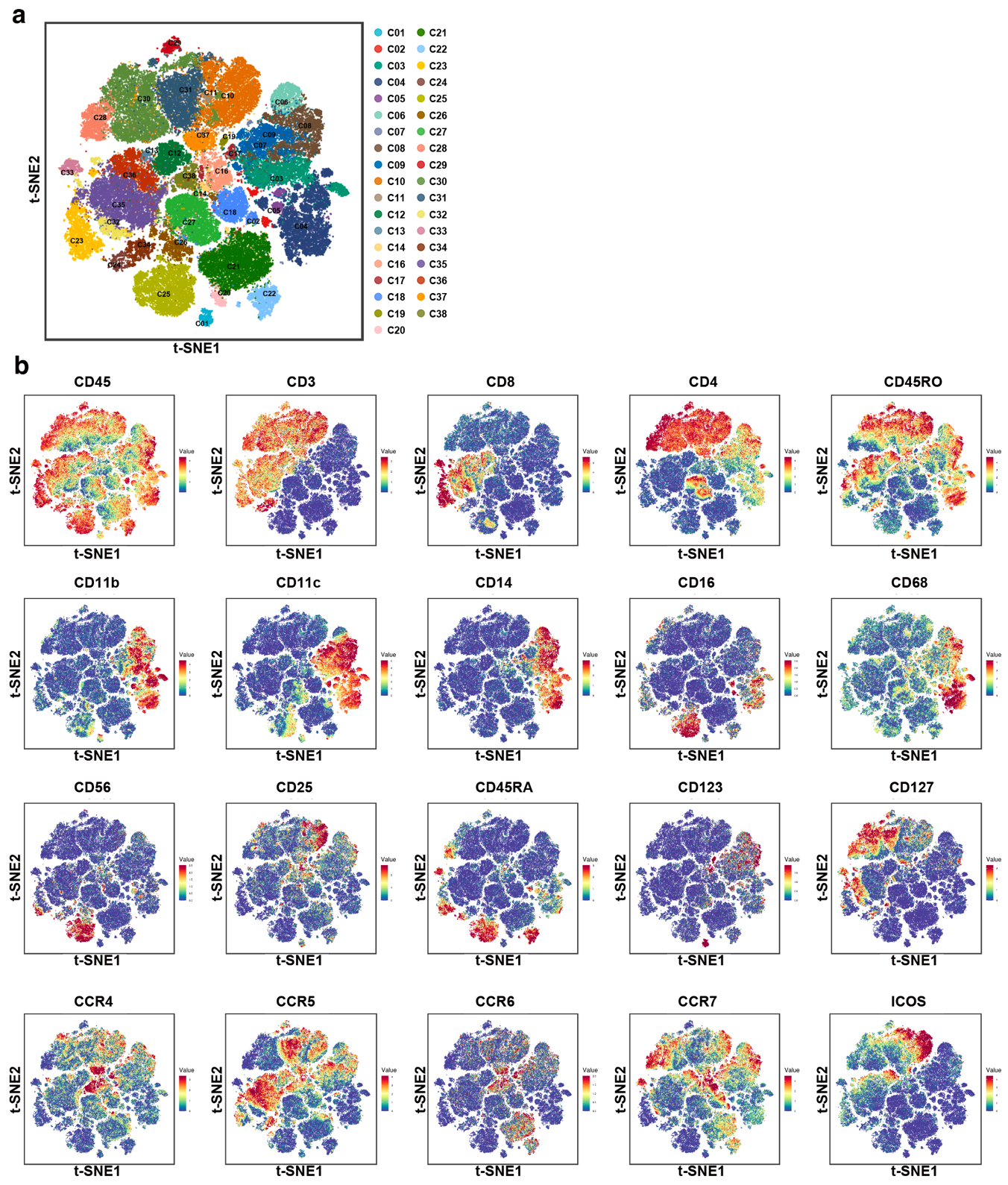
Figure. S2.

**Figure. S2.** **Major immune cell clusters identification.**

**(a)** t-SNE map of the ESCC atlas, color-coded by cell type.

**(b)** Total immune subclass cell identified.

Figure. S3.


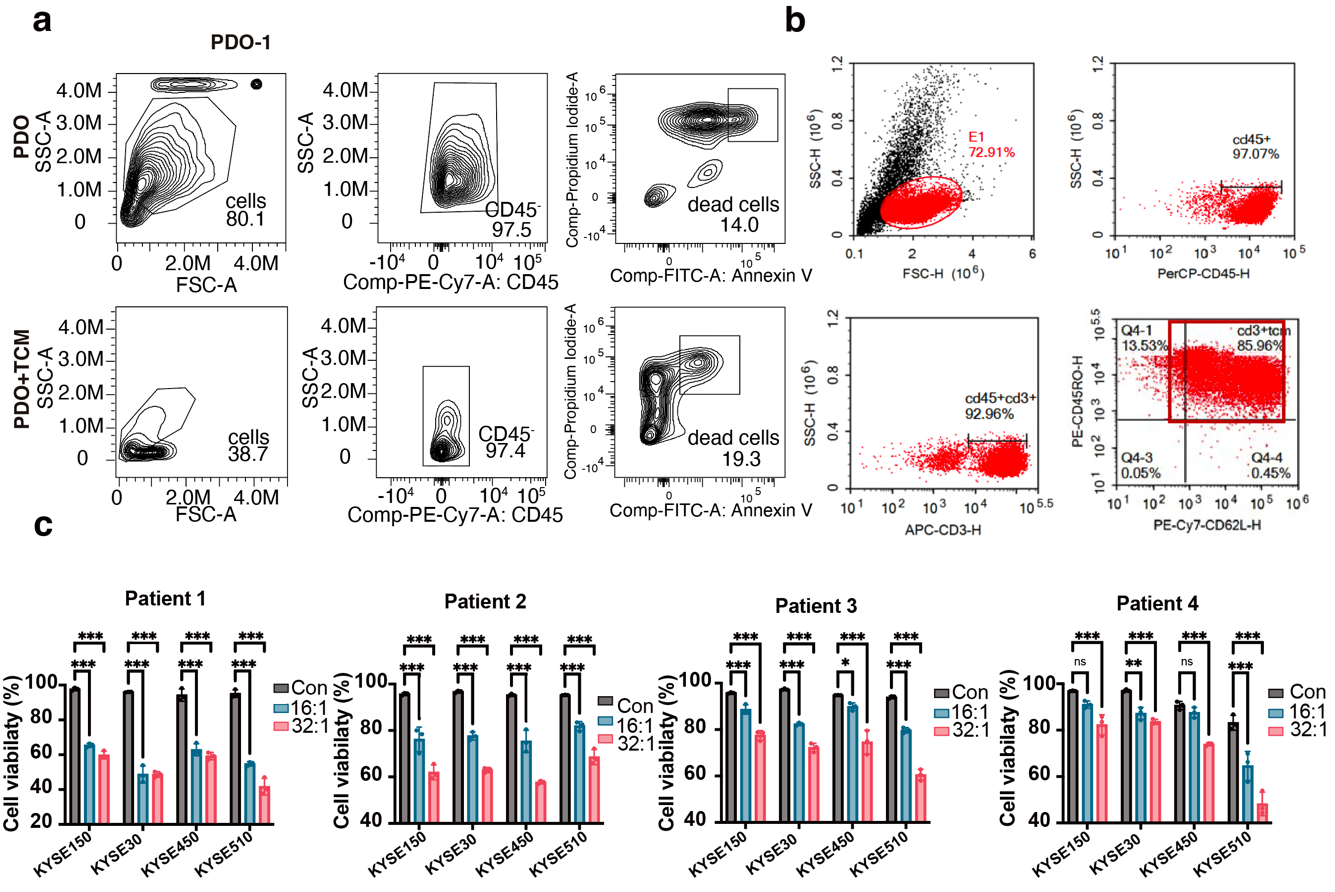


**Figure. S3. Isolation and cytotoxicity of TCM in vitro.**

1. Representative flow cytometry plots showing cell viability of patient-derived organoids (PDOs) cultured alone (PDO, top) or co-cultured with TCM (PDO+TCM, bottom). Cells were gated based on forward scatter (FSC) and side scatter (SSC), followed by exclusion of CD45⁺ immune cells to identify the CD45⁻ tumor population. Dead tumor cells were quantified as Annexin V⁺ and/or PI⁺ within the CD45⁻ gate. All plots use biexponential axes and contour display for improved resolution. Gating was based on population distribution to account for cell-type differences between conditions.
2. To isolate TCM, CD45RO^+^CD3^+^CD62L^+^ cells were selected using flow cytometry.
3. TCM were isolated from four ESCC patients and co-cultured with four ESCC cell lines: KYSE30, KYSE150, KYSE450, and KYSE510. For cytotoxicity assays, TCMs were applied to target cells at effector-to-target (E:T) ratios of 16:1 and 32:1. Cell death was assessed using Annexin V and 7-AAD staining, with subsequent quantification performed via flow cytometry. Killing rate of each treatment group at different effective target ratios. ns: not significant, P < 0.05 (*), P < 0.01 (**), P < 0.001 (***). Mean ± SD, one-way ANOVA test followed by post hoc test with Benjamini–Hochberg correction.

**Figure. S4.**


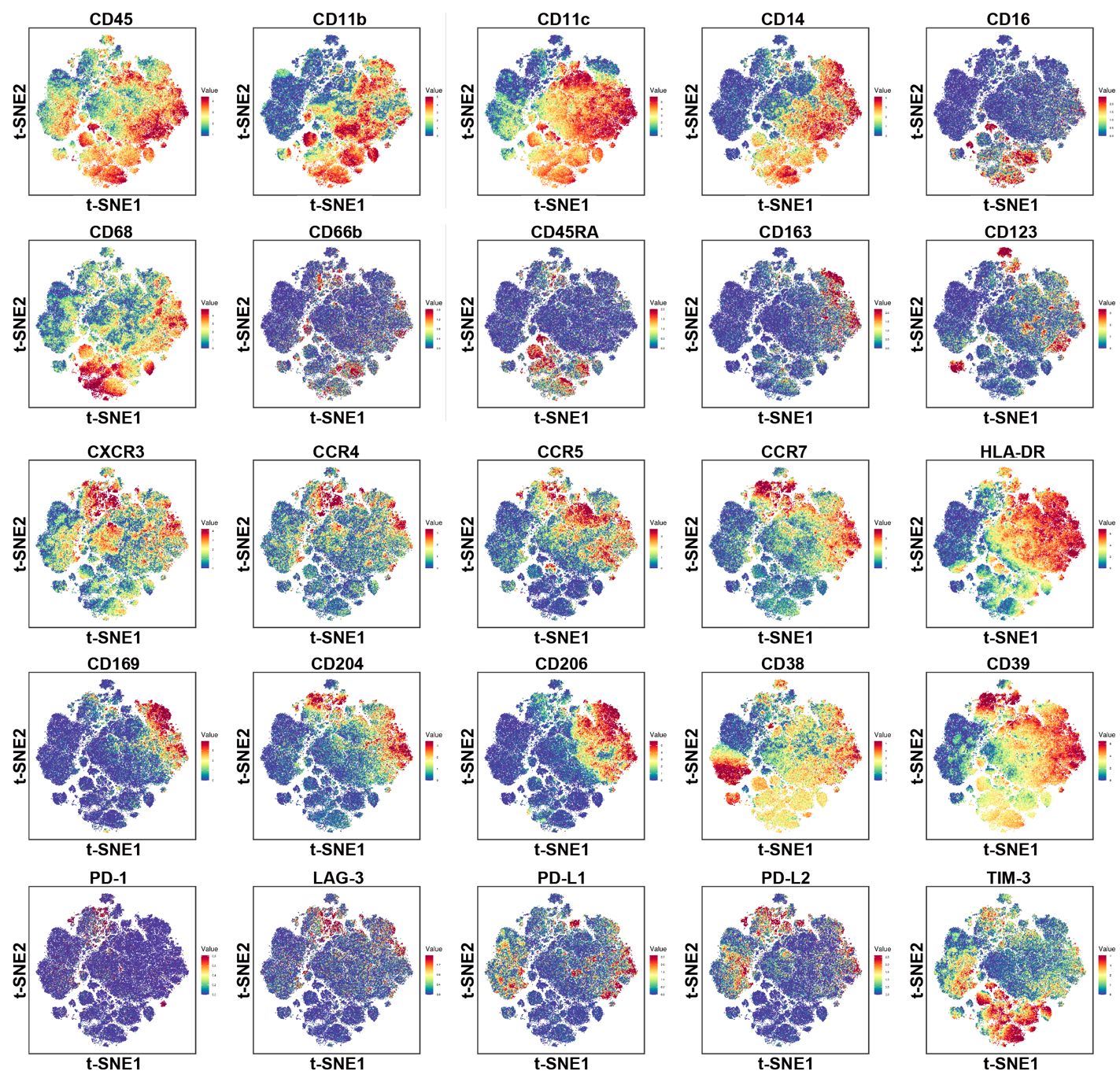


**Figure. S4. Myeloid cell phenotype characterization.** t-SNE map showing the marker expression pattern in myeloid cell.


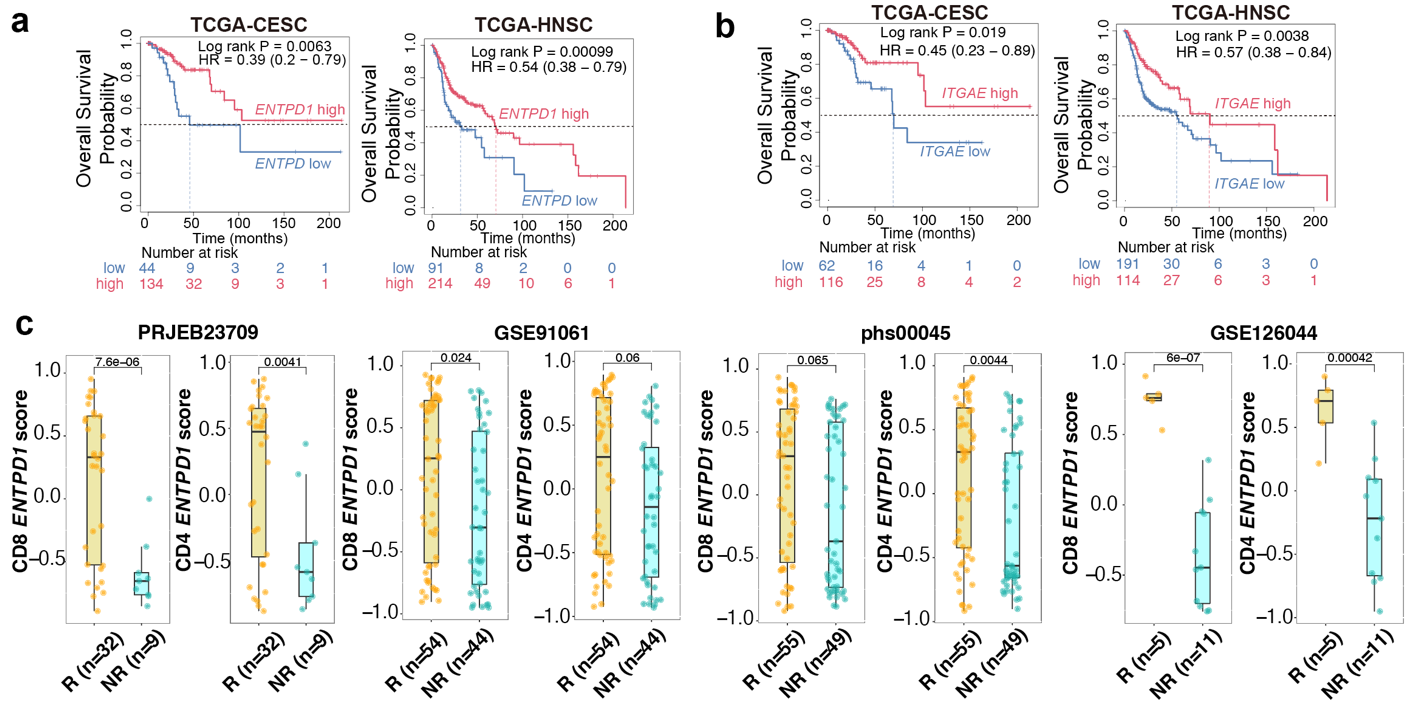
Figure. S5.

**Figure. S5.** The correlation of *ENTPD1* expression with OS and immunotherapy response.

1. Kaplan–Meier plots illustrating the impact of different levels of *ENTPD1* expression within the context of high CD8^+^ T-cell enrichment on OS in patients with cervical squamous cell carcinoma (CESC), and head and neck squamous cell carcinoma (HNSC).

(b) Kaplan–Meier plots illustrating the impact of different levels of ITGAE expression within the context of high CD8^+^ T-cell enrichment on OS in patients with CESC and HNSC.

(c) Analysis of immunotherapy datasets from melanoma (phs000452, PRJEB23709, GSE91061) and NSCLC (GSE126044) cohorts was conducted to assess the enrichment of CD39 (ENTPD1)^+^CD8^+^ and CD39 (ENTPD1)^+^CD4^+^ T cells in patients responding to anti–PD-1 therapy. Box middle lines, median; box limits, upper and lower quartiles; box whiskers, 1.5× the interquartile range (Mann–Whitney *U* test).

**Table S1**.**Clinical characteristics of the ESCC patients.**

| **ID** | **Cancer** | **adjacent nontumor tissues** | **Blood** | **LN metastasis** | **Grade** | **Age** | **Gender** | **Tumor**  **location** | **TNM Stage** |
| --- | --- | --- | --- | --- | --- | --- | --- | --- | --- |
| ESCC_1 | A3-LZH01-H01-T015 | A3-LZH01-H01-T016 | A3-LZH01-H01-B015 | N1+2+3 | G3 | 70 | Male | Lower | T3N1M0 |
| ESCC_2 | A3-LZH01-H01-T017 | A3-LZH01-H01-T018 | A3-LZH01-H01-B017 | N0 | G2 | 75 | Female | Middle | T3N0M0 |
| ESCC_3 | A3-LZH01-H01-T021 | A3-LZH01-H01-T022 | A3-LZH01-H01-B021 | N1+2+3 | G3 | 54 | Female | Middle | T3N2M0 |
| ESCC_4 | A3-LZH01-H01-T023 | A3-LZH01-H01-T024 | A3-LZH01-H01-B023 | N0 | G2 | 61 | Female | Middle | T3N0M0 |
| ESCC_5 | A3-LZH01-H01-T025 | A3-LZH01-H01-T026 | A3-LZH01-H01-B025 | N1+2+3 | G4 | 62 | Male | Lower | T3N3M0 |
| ESCC_6 | A3-LZH01  -H01-T027 | | A3-LZH01-H01-B027 | N0 | G2 | 54 | Male | Middle | T3N0M0 |
| ESCC_7 | A3-LZH01-H01-T029 | A3-LZH01-H01-T030 | A3-LZH01-H01-B029 | N1+2+3 | G2 | 68 | Male | Middle | T2N1M0 |
| ESCC_8 | A3-LZH01-H01-T031 | A3-LZH01-H01-T032 |  | N0 | G2 | 69 | Male | Middle | T3N0M0 |
| ESCC_9 | A3-LZH01-H01-T037 | A3-LZH01-H01-T038 | A3-LZH01-H01-B037 | N1+2+3 | G3 | 59 | Male | Lower | T3N1M0 |
| ESCC_10 | A2-LZH01-H01-T039 | A2-LZH01-H01-T040 | A3-LZH01-H01-B039 | N1+2+3 | G4 | 70 | Male | Lower | T4aN2M0 |
| ESCC_11 | A2-LZH01-H01-T042 | A2-LZH01-H01-T041 | A3-LZH01-H01-B041 | N0 | G4 | 54 | Male | Lower | T3N0M1 |
| ESCC_12 | A2-LZH01-H01-T043 | A2-LZH01-H01-T044 | A3-LZH01-H01-B043 | N0 | G2 | 64 | Male | Middle | T3N0M0 |
| ESCC_13 | A2-LZH01-H01-T045 | A2-LZH01-H01-T046 |  | N1+2+3 | G3 | 65 | Male | Lower | T3N2M0 |
| ESCC_14 | A2-LZH01-H01-T047 | A2-LZH01-H01-T048 | A3-LZH01-H01-B047 | N1+2+3 | G3 | 54 | Male | Lower | T2N2M0 |
| ESCC_15 | A2-LZH01-H01-T053 | A2-LZH01-H01-T052 | A3-LZH01-H01-B052 | N1+2+3 | G2 | 70 | Male | Lower | T1bN1M0 |
| ESCC_16 | A2-LZH01-H01-T056 | A2-LZH01-H01-T057 | A3-LZH01-H01-B056 | N1+2+3 | G3 | 77 | Male | Middle | T3N2M0 |
| ESCC_17 | A2-LZH01-H01-T054 | A2-LZH01-H01-T055 | A3-LZH01-H01-B054 | N0 | G2 | 63 | Male | Upper | T2N0M0 |
| ESCC_18 | A3-LZH01-H01-T058 | A3-LZH01-H01-T059 | A3-LZH01-H01-B058 | N0 | G2 | 73 | Male | Lower | T2N0M0 |
| ESCC_19 | A3-LZH01-H01-T060 | A3-LZH01-H01-T061 | A3-LZH01-H01-B060 | N0 | G2 | 65 | Female | Lower | T3N0M0 |
| ESCC_20 | A3-LZH01-H01-T065 | A3-LZH01-H01-T066 | A3-LZH01-H01-B065 | N1+2+3 | G3 | 68 | Male | Lower | T3N1M0 |
| ESCC_21 | A3-LZH01-H01-T067 | A3-LZH01-H01-T068 | A3-LZH01-H01-B067 | N0 | G2 | 14 | Female | Upper | T3N0M0 |
| ESCC_22 | A3-LZH01-H01-T073 | A3-LZH01-H01-T074 | A3-LZH01-H01-B073 | N1+2+3 | G3 | 79 | Male | Lower | T3N0M0 |
| ESCC_23 | A3-LZH01-H01-T071 | A3-LZH01-H01-T072 | A3-LZH01-H01-B071 | N1+2+3 | G3 | 78 | Female | Middle | T3N2M0 |
| ESCC_24 | A3-LZH01-H01-T070 | A3-LZH01-H01-T069 | A3-LZH01-H01-B069 | N0 | G2 | 76 | Male | Middle | T3N0M0 |
| ESCC_25 | A3-LZH01-H01-T075 | A3-LZH01-H01-T076 | A3-LZH01-H01-B075 | N1+2+3 | G2 | 55 | Male | Middle | T2N2M0 |

Table S2. All antibodies and corresponding clone, provider, and metal tag.

| **List** | **Metal tag** | **Antibody** | **Clone number** | **Company** | **Catalog number** |
| --- | --- | --- | --- | --- | --- |
| 1 | 89Y | CD45 | HI30 | Biolegend | 304002 |
| 2 | 115In | CD3 | UCHT1 | Biolegend | 300438 |
| 3 | 139La | CD68 | Y1/82A | Biolegend | 333802 |
| 4 | 141Pr | CD56 | NCAM16.2 | BD | 559043 |
| 5 | 142Nd | CD19 | HIB19 | Biolegend | 302268 |
|  | 142Nd | TCR γ/δ | 5A6.E9 | PLT | 100P001A |
| 6 | 143Nd | CD196 (CCR6) | G034E3 | Biolegend | 353402 |
| 7 | 144Nd | CD14 | M5E2 | Biolegend | 301862 |
| 8 | 145Nd | CD45RA | HI100 | Biolegend | 304102 |
| 9 | 146Nd | CD123 (IL-3Rα) | 6H6 | Biolegend | 306002 |
| 10 | 147Sm | CD66b | G10F5 | Biolegend | 305102 |
| 11 | 148Nd | CD194 (CCR4) | L291H4 | Biolegend | 359402 |
| 12 | 149Sm | CD169 (Siglec-1) | 7-239 | Biolegend | 346002 |
| 13 | 150Nd | CD223 (LAG-3) | 874501 | RD | MAB23193 |
| 14 | 151Eu | CD38 | HIT2 | Biolegend | 303502 |
| 15 | 152Sm | CD195 (CCR5) | J418F1 | Biolegend | 359102 |
| 16 | 153Eu | CD274 (PD-L1) | 29E.2A3 | Biolegend | 329716 |
| 17 | 154Sm | CD163 | GHI/61 | Biolegend | 333602 |
| 18 | 155Gd | CD206 (MMR) | 15-2 | Biolegend | 321150 |
| 19 | 156Gd | CD204 (SR-AI) | 351615 | RD | MAB2708 |
| 20 | 157Gd | CD39 | A1 | Biolegend | 328202 |
| 21 | 158Gd | CD197 (CCR7) | G043H7 | Biolegend | 353256 |
| 22 | 159Tb | CD11c | Bu15 | Biolegend | 337202 |
| 23 | 160Gd | CD25 (IL-2Rα) | 24212 | RD | MAB1020 |
| 24 | 161dy | CD152 (CTLA-4) | L3D10 | Biolegend | 349902 |
| 25 | 162Dy | FOXP3 | PCH101 | eB | 14-4776-82 |
| 26 | 163Dy | CD183 (CXCR3) | G025H7 | Biolegend | 353750 |
| 27 | 164Dy | RORγ (RORC) | 600214 | RD | MAB6109 |
| 28 | 165Ho | CD366 (Tim-3) | F38-2E2 | Biolegend | 345010 |
| 29 | 166Er | perforin | B-D48 | Abcam | ab47225 |
| 30 | 167Er | CD278 (ICOS) | C398.4A | Biolegend | 313502 |
| 31 | 168Er | T-bet | 4B10 | Biolegend | 644802 |
| 32 | 169Tm | CD45RO | UCHL1 | Biolegend | 304202 |
| 33 | 170Er | CD127 (IL-7Rα) | A019D5 | Biolegend | 351302 |
| 34 | 171Yb | Gata-3 | TWAJ | eB | 14-9966-82 |
| 35 | 172Yb | CD273 (PD-L2) | 24F.10C12 | Biolegend | 329623 |
| 36 | 173Y | Granzyme B Recombinant | QA16A02 | Biolegend | 372202 |
| 37 | 174Yb | CD279 (PD-1) | EH12.2H7 | Biolegend | 329926 |
| 38 | 175Lu | CD16 | 3G8 | Biolegend | 302057 |
| 39 | 176Yb | HLA-DR | L243 | Biolegend | 307648 |
| 40 | 197Au | CD4 | RPA-T4 | Biolegend | 300541 |
| 41 | 198Pt | CD8a | RPA-T8 | Biolegend | 301074 |
| 42 | 209Bi | CD11b | M1/70 | Biolegend | 101202 |

Table S3. qRT-PCR primers.

| **List** | **Primer** | **Sequence (5’-3’)** |
| --- | --- | --- |
| 1 | M-iNOS-F | GTTCTCAGCCCAACAATACAAGA |
| 2 | M-iNOS-R | GTGGACGGGTCGATGTCAC |
| 3 | M-IL-4-F | GGTCTCAACCCCCAGCTAGT |
| 4 | M-IL-4-R | GCCGATGATCTCTCTCAAGTGAT |
| 5 | M-ARG1-F | TGGCTTGCGAGACGTAGAC |
| 6 | M-ARG1-R | GCTCAGGTGAATCGGCCTTTT |
| 7 | M-IL-12-F | ACTCTGCGCCAGAAACCTC |
| 8 | M-IL-12-R | CACCCTGTTGATGGTCACGAC |
| 9 | M-GAPDH-F | AGGTCGGTGTGAACGGATTTG |
| 10 | M-GAPDH-R | TGTAGACCATGTAGTTGAGGTCA |
| 11 | M-CD206-F | CTCTGTTCAGCTATTGGACGC |
| 12 | M-CD206-R | TGGCACTCCCAAACATAATTTGA |
| 13 | M-CD274-F | GCTCCAAAGGACTTGTACGTG |
| 14 | M-CD274-R | TGATCTGAAGGGCAGCATTTC |
| 15 | M-IL-1β-F | GAAATGCCACCTTTTGACAGTG |
| 16 | M-IL-1β-R | TGGATGCTCTCATCAGGACAG |
| 17 | M-PDCD1-F | CAGCTTGTCCAACTGGTCG |
| 18 | M-PDCD1-R | GCTCAAACCATTACAGAAGGCG |
| 19 | M-CD39-F | AAGGTGAAGAGATTTTGCTCCAA |
| 20 | M-CD39-R | GCATCCAACACAATCCCATACT |
| 21 | M-Tim3-F | TCAGGTCTTACCCTCAACTGTG |
| 22 | M-Tim3-R | GGCATTCTTACCAACCTCAAACA |
| 23 | M-GZMB-F | TCTCGACCCTACATGGCCTTA |
| 24 | M-GZMB-R | TCCTGTTCTTTGATGTTGTGGG |
| 25 | M-IFNG-F | GCCACGGCACAGTCATTGA |
| 26 | M-IFNG-R | TGCTGATGGCCTGATTGTCTT |

Table S4. Clinical Information for PDO.

| **ID** | **Age** | **Gender** | **Tumor** |
| --- | --- | --- | --- |
| PDO_1 | 57 | Male | ESCC |
| PDO_2 | 51 | Male | ESCC |

**Table S5. Clinical information.**

| **ID** | **Age** | **Gender** | **Location** | **Grade** | **TNM Stage** | | | |
| --- | --- | --- | --- | --- | --- | --- | --- | --- |
| 1 | 81 | Male | Middle | G3 | T3 | N0 | M0 | II |
| 2 | 55 | Male | Upper | G2 | T0 | N1 | M0 | III |
| 3 | 52 | Male | Middle | G2 | T4a | N1 | M0 | IV |
| 4 | 45 | Male | Middle | G2 | T1b | N0 | M0 | I |
